# Apigenin ameliorates postoperative inflammation and accelerates wound healing in a rat model of perianal abscess by targeting the JAK1/STAT3 pathway in macrophages

**DOI:** 10.3389/fimmu.2026.1726917

**Published:** 2026-01-28

**Authors:** Ruixue Dai, Liangliang Cao, Jiahui Ren, Jingru Liu, Kexin Zhu, Peidong Chen, Weifeng Yao, Xuelong Zhao, Beihua Bao, Li Zhang

**Affiliations:** 1 Department of Pharmacy, Nanjing Hospital of Chinese Medicine affiliated to Nanjing University of Chinese Medicine, Nanjing, China; 2 Jiangsu Collaborative Innovation Centre of Chinese Medicinal Resources Industrialization, National and Local Collaborative Engineering Centre of Chinese Medicinal Resources Industrialization and Formulae Innovative Medicine, Nanjing, China; 3 School of Pharmacy, Nanjing University of Chinese Medicine, Nanjing, China; 4 Nanjing University of Chinese Medicine Hanlin College, Taizhou Key Laboratory of Traditional Chinese Medicine and Comprehensive Health Products Development, Taizhou Engineering Research Center for Quality and Industrialization of Traditional Chinese Medicine, Taizhou, China

**Keywords:** anti-inflammatory, apigenin, *Cayratia japonica* ointment, JAK1/STAT3 signaling pathway, perianal abscess surgery, wound healing

## Abstract

**Background:**

Excessive inflammation mediated by macrophages impedes wound healing following surgical drainage of perianal abscess, a common yet challenging condition in colorectal surgery. *Cayratia japonica* ointment (CJO) has shown clinical efficacy, but its active immunomodulatory constituents and mechanisms remain unclear.

**Methods:**

We evaluated the anti-inflammatory effects of seven CJO-derived flavonoids in LPS-stimulated RAW264.7 macrophages. Skin pharmacokinetics were assessed in mice, and therapeutic efficacy was tested in a rat postoperative perianal abscess model. Molecular mechanisms were investigated via Western blot, RT-qPCR, and molecular docking.

**Results:**

Pharmacokinetic analysis revealed that apigenin exhibited the highest transdermal exposure among all detected components. *In vitro*, apigenin significantly suppressed LPS-induced production of IL-6, TNF-α, and IL-1β in RAW 264.7 cells. *In vivo*, apigenin treatment markedly reduced serum levels of these pro-inflammatory cytokines, attenuated inflammatory cell infiltration, and promoted collagen deposition at the wound site. Mechanistically, apigenin downregulated both the phosphorylation and total protein levels of JAK1 and STAT3, suggesting functional modulation of the JAK1/STAT3 signaling pathway.

**Conclusion:**

Apigenin may contribute to observed biological effects of CJO that promotes wound healing by attenuating macrophage-driven inflammation through potential modulation of the JAK1/STAT3 signaling pathway, offering a promising natural immunomodulator for postoperative wound management.

## 1 Introduction

Perianal abscess is a common acute general surgical condition, with an estimated incidence of approximately 2 per 10,000 individuals (1). It causes significant pain and discomfort, markedly impairing patients’ quality of life (2). Surgical incision and drainage remains the standard treatment (3). However, the procedure often results in extensive tissue trauma, and the open wounds are highly prone to secondary infection. Postoperative complications—such as persistent pain, edema, and delayed healing—are common, leading to suboptimal recovery and prolonged hospitalization (4). Accumulating evidence underscores that dysregulated innate immune responses—particularly sustained activation of macrophages and overproduction of pro-inflammatory cytokines such as tumor necrosis factor-alpha (TNF-α), interleukin-1 beta (IL-1β), and IL-6—are central to impaired tissue repair in chronic wounds (5, 6).

Wound healing is a highly orchestrated process involving dynamic interactions among inflammatory cells, fibroblasts, endothelial cells, and extracellular matrix components. It progresses through overlapping phases—hemostasis, inflammation, proliferation, and remodeling—each tightly regulated by cytokines, growth factors, and signaling pathways (7, 8). Macrophages are key mediators of the inflammatory response in wound healing. Their excessive activation drives sustained release of pro-inflammatory cytokines (e.g., IL-6, TNF-α), which disrupts the normal healing cascade and promotes chronic inflammation (9). Persistent activation of JAK1/STAT3 signaling in macrophages has been shown to amplify this cytokine production, contributing to impaired tissue repair (10). Consequently, therapeutic strategies that fine-tune macrophage function without compromising host defense are highly desirable.

Natural products, especially flavonoids, have emerged as promising immunomodulators due to their multi-target anti-inflammatory properties and favorable safety profiles (11). *Cayratia japonica* (Thunb.) Gagnep., a member of the Vitaceae family, has been used in Traditional Chinese Medicine (TCM) for centuries under the name “Wulianmei” (first documented in the Tang Materia Medica) for its heat-clearing, detoxifying, and anti-edema properties (12). It is traditionally prescribed for boils, carbuncles, erysipelas, and inflammatory conditions. Based on decades of clinical experience at the National Anorectal Center of Nanjing Hospital of Chinese Medicine, a topical preparation—*Cayratia japonica* ointment (CJO)—has been employed for over 50 years to manage postoperative perianal wounds, demonstrating favorable outcomes (13), yet the active constituents and underlying immunological mechanisms remain elusive. Our prior work identified flavonoids as the major skin-retained components of CJO (14), raising the question of which specific compound drives its anti-inflammatory efficacy.

Among these, apigenin—a naturally occurring flavone—has demonstrated potent anti-inflammatory effects in various models by modulating NF-κB, MAPK, and JAK/STAT signaling (15–17). Notably, the JAK1/STAT3 pathway is a key regulator of cytokine production in macrophages and has been implicated in chronic inflammatory skin conditions (18). This pathway is particularly relevant in perianal abscess and postoperative wounds, as it mediates inflammation amplification and tissue repair imbalance in chronic inflammatory skin injuries. However, whether apigenin contributes to CJO’s wound-healing effects via JAK1/STAT3 modulation in the context of perianal abscess remains unexplored.

Here, we integrate *in vitro* immunopharmacology, *in vivo* efficacy, skin pharmacokinetics, and molecular docking to demonstrate that apigenin may contribute to observed biological effects of CJO. Our data suggest that apigenin achieves high skin exposure after topical application and effectively suppresses macrophage-mediated inflammation by potential modulation of the JAK1/STAT3 pathway, thereby accelerating wound closure. Our findings position apigenin as a naturally derived and mechanism-validated, single-component alternative to complex herbal formulations for immunomodulatory wound therapy.

## 2 Materials and methods

### 2.1 Reagents and materials

*Cayratia japonica* (Batch No. 221101) was obtained from Anhui Huchuntang Traditional Chinese Medicine Tea Slice Co., Ltd. (Anhui, China). Vaseline Yellow (Batch No. A2220185) was purchased from Shanghai Aladdin Biochemical Technology Co., Ltd. (Shanghai, China). Reference standards—including esculetin, schaftoside, luteolin-7-O-glucuronide, luteolin-7-O-glucoside, apigenin-7-O-glucuronide, luteolin, apigenin, and chrysin (all ≥98% purity by HPLC)—were sourced from Shanghai Yuanye Biotechnology Co., Ltd. (Shanghai, China). Digoxin (internal standard, ≥98% purity; Batch No. DST210420-086) was provided by Chengdu Desite Biotechnology Co., Ltd. (Chengdu, China). HPLC-grade methanol and acetonitrile (Merck KGaA, Darmstadt, Germany), formic acid (Thermo Fisher Scientific, Waltham, MA, USA), and the Pierce™ BCA Protein Assay Kit (Cat. No. 23227) were used for sample preparation and protein quantification. Lipopolysaccharide (LPS, Cat. No. L2630) was purchased from Sigma-Aldrich (St. Louis, MO, USA). The IL-6 ELISA Kit (Specification: 96T; Catalog No. RX203049M), The TNF-α ELISA Kit (Specification: 96T; Catalog No. RX202412M) and the IL-1β ELISA Kit (Specification: 96T; Catalog No. RX203063M) were purchased from Quanzhou Ruixin Biotechnology Co., Ltd. (Quanzhou, China). The RNA-easy Isolation Reagent (Catalog No. R701-01), HiScript II 1st Strand cDNA Synthesis Kit (Catalog No. R211-01), and ChamQ SYBR qPCR Master Mix (Low ROX Premixed) (Catalog No. Q331-02) were purchased from Nanjing Novogen Biotechnology Co., Ltd. (Nanjing, China). JAK1 (Catalog No. 66466-1-Ig, 1:2000), STAT3 (Catalog No. 10253-2-AP, 1:2000), p-STAT3 (Ser727) (Catalog No. 28945-1-AP, 1:1000), GAPDH (Catalog No. 10494-1-AP, 1:5000), HRP-conjugated Affinipure Goat Anti-Rabbit IgG (H+L) (Catalog No. SA00001-2, 1:5000), and HRP-conjugated Affinipure Goat Anti-Mouse IgG (H+L) (Catalog No. SA00001-1, 1:5000) were purchased from Proteintech (Wuhan, China). P-JAK1 (Tyr1022) [Tyr1034] (Catalog No: #AF2012, 1:1000) was purchased from Affinity (Melbourne, Australia).

### 2.2 Cell culture and treatment

RAW264.7 murine macrophages (Shanghai Zhongqiao Xinzhuo Biotechnology Co., Ltd.) were maintained in high-glucose DMEM supplemented with 10% fetal bovine serum and 1% penicillin–streptomycin at 37°C under 5% CO_2_. For cytotoxicity assessment, cells (3 × 10^4^ cells/well in 96-well plates) were treated with seven flavonoids (0–4 μM) for 24 h, followed by CCK-8 assay (absorbance at 450 nm (19).

To establish an inflammatory model, RAW264.7 cells were stimulated with LPS (0–1000 ng/mL). Based on dose–response ELISA results for IL-6, TNF-α, and IL-1β, 500 ng/mL LPS was selected for subsequent experiments. Cells (6 × 10^5^/well in 6-well plates) were pretreated with flavonoids (0.1, 0.5, or 2.5 μM) for 2 h, then co-incubated with LPS (500 ng/mL) for 24 h. Supernatants were collected (3000 rpm, 10 min) and analyzed for cytokines using ELISA kits per manufacturer instructions (20).

### 2.3 Animals and treatment

Pharmacokinetic experiments were conducted in accordance with relevant literature (21, 22). ICR mice (30 ± 5 g, equal sex ratio; Hangzhou Ziyuan Experimental Animal Co., Ltd., SCXK(Su)2018-0049) were used for pharmacokinetic studies. After anesthetizing with an intraperitoneal injection of sodium pentobarbital (50 mg/kg), the back hair was shaved (using depilatory cream on areas that were not cleanly shaved), the area for drug application on the mouse’s back was marked with a marker (d=2 cm), ointment (500 mg) was applied, and the area was covered with gauze for bandaging. At predetermined time points (0.5–12 h, n = 8/time), mice were euthanized by intraperitoneal injection of sodium pentobarbital (150 mg/kg). Skin samples from the application site were collected immediately after confirmation of death, weighed, and stored at −80°C.

The effects of apigenin on postoperative wounds in a model of perianal abscess surgery in SD rats were evaluated with reference to relevant literature (23). A fecal-contaminated perianal wound model was employed to simulate severe local infection and inflammation, rather than to fully recapitulate the epithelialized sinus tract of human anal fistula. Male Sprague-Dawley rats (200–220 g; Jiangsu Huachuang Xinno, SCXK(Su)2020-0009) were acclimatized for 7 days. Rats were anesthetized by intraperitoneal injection of sodium pentobarbital (35 mg/kg). A 2-cm full-thickness circular wound was created on the dorsum using a sterile biopsy punch. A fecal suspension (prepared by homogenizing 10 g fresh rat feces in 5 mL saline, centrifuged at 3000 rpm for 15 min) was applied (1 mL/wound). After 48 h, purulent exudate and foul odor confirmed successful model establishment. The successfully modeled rats were randomly divided into 6 groups (n=12): model group (M, Vaseline Yellow); positive drug group (P, recombinant human epidermal growth factor gel, 10 µg/g recombinant human epidermal growth factor - loaded gel, administered at 300 mg gel/wound/day); low dose apigenin group (AL, 17.72 µg/g apigenin - loaded ointment, administered at 300 mg ointment/wound/day); high dose apigenin group (AH, 70.88 µg/g apigenin - loaded ointment, administered at 300 mg ointment/wound/day); high dose of *Cayratia japonica* extract group (CEH, 61.19 mg/g *Cayratia japonica* extract - loaded ointment, administered at 300 mg ointment/wound/day); high dose *Cayratia japonica* powder group (CPH, 0.60 g/g *Cayratia japonica* powder - loaded ointment, administered at 300 mg ointment/wound/day).

A blank control (BK) group underwent no surgery or treatment. After successful modeling, treatments were applied daily. Fresh fecal supernatant (1 mL) was reapplied 30 min before each dressing change to sustain infection.

The animal study was approved by the Animal Ethics Committee of the Nanjing University of Chinese Medicine (Ethics Approval Number: 202410A017). The testing process followed the Guide for the Care and Use of Laboratory Animals. All animals were housed in the SPF-level laboratory animal center at Nanjing University of Chinese Medicine. Animals were housed under SPF conditions (25 ± 3°C, 55 ± 10% humidity, 12-h light/dark cycle).

### 2.4 HPLC-MS/MS analysis

**Figure 1:**
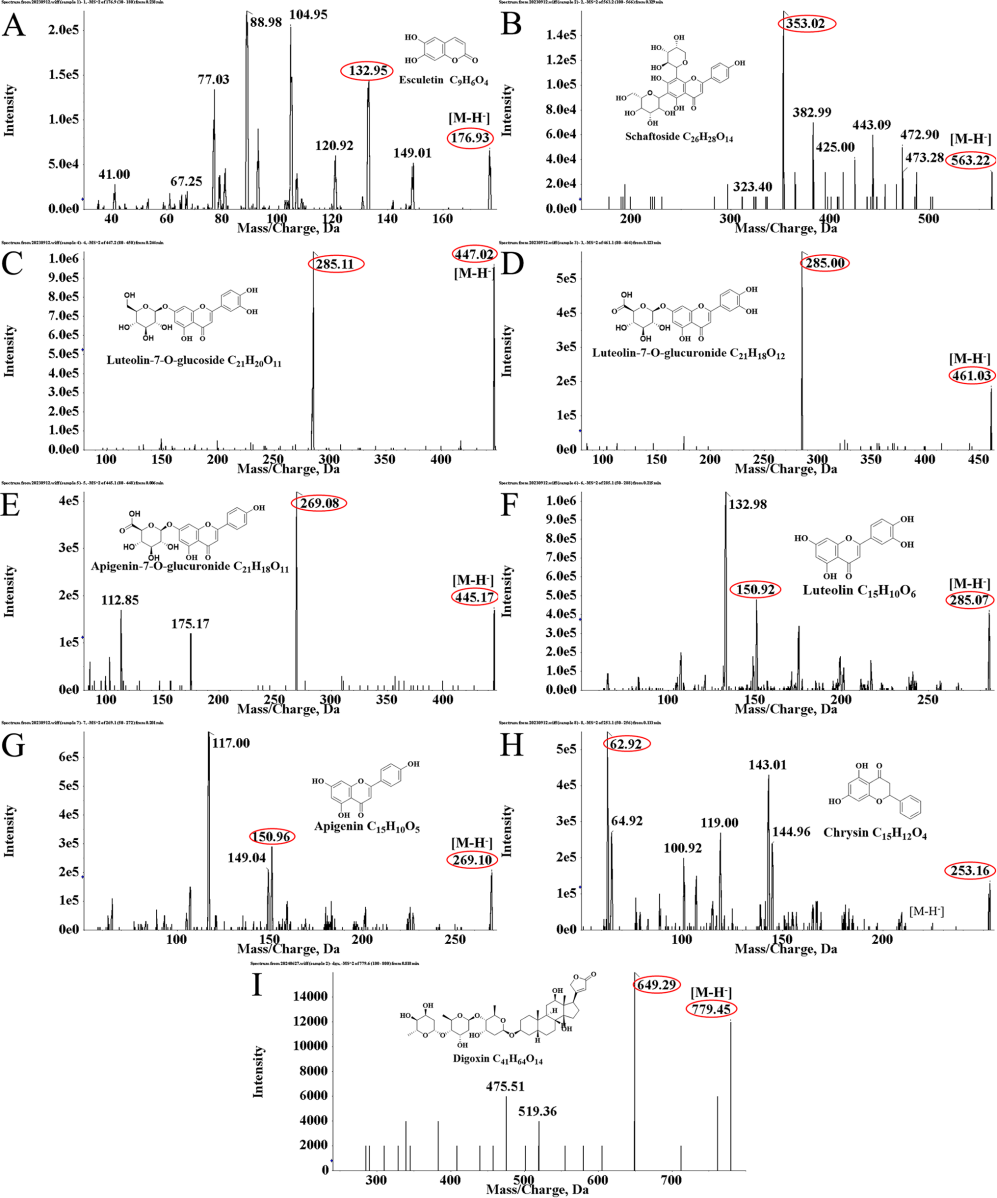
Q1 (*m/z*) and Q3 (*m/z*) transitions of eight analytes and internal standards from *Cayratia japonica* (CJ) in MS/MS spectra. **(A)** Esculetin. **(B)** Schaftoside. **(C)** Luteolin-7-O-glucoside. **(D)** Luteolin-7-O-glucuronide. **(E)** Apigenin-7-O-glucuronide. **(F)** Luteolin. **(G)** Apigenin. **(H)** Chrysin. **(I)** Digoxin.

Chromatographic separation was performed on a Shimadzu UPLC system (LC-20AD XR) equipped with an Xtimate^®^ C18 column (2.1 × 50 mm, 3.0 μm) at 40°C. Mass spectrometry was conducted on an AB Sciex QTRAP 5500 system in ESI-MRM mode. The MRM transitions for the seven flavonoids and internal standard (digoxin) are listed in **Figures 1A–I**. The method was validated for selectivity, linearity (r² > 0.99), precision (RSD < 15%), accuracy (85–115%), matrix effect, and stability per FDA bioanalytical guidelines (24) (Detailed method validation data are provided in **Supplementary Tables S1**-**S6**).

### 2.5 Wound healing assessment

On days 7 and 14 post-modeling (n = 3/group), wound tissues were harvested, fixed in 4% paraformaldehyde, embedded in paraffin, and sectioned (5 μm). Sections were stained with hematoxylin–eosin (H&E) and Masson’s trichrome to evaluate inflammation, re-epithelialization, and collagen deposition (25).

Histopathological quantification was performed on blinded samples. For H&E-stained sections, inflammatory cell infiltration was assessed by manually counting nucleated immune cells within the wound bed across five random high-power fields (HPF, 400×) per section. For Masson’s trichrome–stained sections, collagen deposition was quantified as the percentage of blue-stained area relative to total wound tissue area using color thresholding in ImageJ (v1.53k). All analyses were performed independently by two investigators (26, 27).

Quantitative histopathological analysis was performed by two blinded observers using ImageJ 1.53t. For H&E sections, five randomly selected fields (400× magnification; 0.15 mm²/field) per wound section were analyzed for inflammatory cell density (cells/mm²) using color deconvolution and automated particle counting (size threshold: 5-20 μm²; circularity: 0.5-1.0). For Masson’s trichrome sections, collagen deposition was quantified as the percentage of blue-stained area relative to total tissue area using threshold-based color segmentation (Hue: 180-230; Saturation: 10-255; Brightness: 0-180). Inter-observer correlation coefficient (ICC) was 0.92 for cell counting and 0.89 for collagen analysis. Data represent mean ± SEM from 6 animals per group, with 3 sections analyzed per animal.

### 2.6 Serum inflammatory cytokine assay

Blood was collected from the abdominal aorta on days 7 and 14 (n = 6/group), centrifuged (3000 rpm, 15 min). Then, TNF-α, IL-6, and IL-1β levels were quantified using ELISA kits.

### 2.7 Western blot analysis

Total protein was extracted from wound tissues using RIPA buffer with protease/phosphatase inhibitors. Protein concentration was determined by BCA assay. Equal amounts (30 μg) were separated by 10% SDS-PAGE and transferred to PVDF membranes (Millipore, IPVH00010). After blocking with 5% non-fat milk, membranes were incubated overnight at 4°C with primary antibodies (JAK1, p-JAK1, STAT3, p-STAT3, GAPDH), followed by HRP-conjugated secondary antibodies (1 h, RT). Bands were visualized using ECL reagent (Shanghai Tianeng, 180-5001) and quantified by ImageJ. Target protein expression was normalized to GAPDH (28).

Each detection index included 3 biological replicates, and data were derived from 3 independent experiments. Full-length blots are provided in Supplementary materials B.

### 2.8 Real-time quantitative PCR

Total RNA was extracted using RNA-easy Isolation Reagent and reverse-transcribed into cDNA using the HiScript II 1st Strand cDNA Synthesis Kit. qPCR was performed using using the ChamQ SYBR qPCR Master Mix (Low ROX Premixed) on an Applied Biosystems™ 7500 real-time PCR system (Thermo). Primer sequences are listed as follows: JAK1 (forward): 5′-TCTGTTTGCTCAGGGACAGT-3′, JAK1 (reverse): 5′-AGCCATCCCTAGACACTCGT-3′; STAT3 (forward): 5′- ATCACGCCTTCTACAGACTGC-3′, STAT3 (reverse): 5′- CATCCTGGAGATTCTACCACT-3′; GAPDH (forward): 5′- CCTGGAGAAACCTGCCAAGTAT-3′, GAPDH (reverse): 5′- TAGCCCAGGATGCCCTTTAGT-3′. All primers were rat-specific for JAK1 (NCBI RefSeq: XM_006238452.2) and STAT3 (NCBI RefSeq: NM_012747.2). Melt curve analysis confirmed single-peak amplification profiles for all primer pairs, demonstrating absence of primer-dimers and non-specific products. Cycling condition: 95°C for 30 s for pre-denaturation; then, the denaturation stage at 95°C for 10 s, and annealing at 60°C for 30 s, for a total of 40 cycles; the melt curve consisted of 95°C for 15 s, 60°C for 60 s, and 95°C for 15 s. Gene expression was calculated by the 2^−ΔΔCt^ method, normalized to GAPDH.

Each sample included 3 biological replicates, and data were derived from 3 independent experiments.

### 2.9 Molecular docking

Molecular docking was performed using AutoDock Tools 1.5.7. The 3D structures of JAK1 (PDB ID: 6N7A) and STAT3 (PDB ID: 6QHD) were retrieved from the RCSB Protein Data Bank and prepared using PyMOL 2.6 by removing water molecules and adding polar hydrogen atoms. The 3D structure of apigenin was downloaded from PubChem (CID: 5280443) and geometry-optimized via Gaussian 09 at the B3LYP/6-31G(d) level of theory. Docking was conducted with a grid box size of 60×60×60 Å and grid spacing of 0.375 Å, centered on the active sites of JAK1 (ARG-1007, ASP-921, ASP-1003, GLU-925, HIS-918) and STAT3 (ARG-609, SER-611, LYS591). Docking simulations were run with 100 Lamarckian genetic algorithm runs, and the best-scoring pose was selected based on binding energy and interaction consistency. Tofacitinib and chrysin were employed as positive and negative controls, respectively.

### 2.10 Justification for the sample size

Sample sizes were determined based on prior studies in rodent wound models (25) and pilot experiments, aiming to balance statistical reliability with ethical animal use. A sample size of n = 6 per group was estimated to provide >80% power to detect a ≥40% difference in primary outcomes (e.g., wound closure, IL-6) at α = 0.05, consistent with effect sizes reported in similar preclinical studies.

### 2.11 Statistical analysis

Pharmacokinetic parameters were calculated using non-compartmental analysis (DAS 2.0). Data are expressed as mean ± SD. Statistical comparisons were performed using one- or two-way ANOVA followed by Tukey’s *post hoc* test (SPSS 25.0, GraphPad Prism 9.0). *p* < 0.05 or *p* < 0.01 was considered statistically significant.

## 3 Results

### 3.1 *In vitro* effects of seven flavonoids

**Figure 2:**
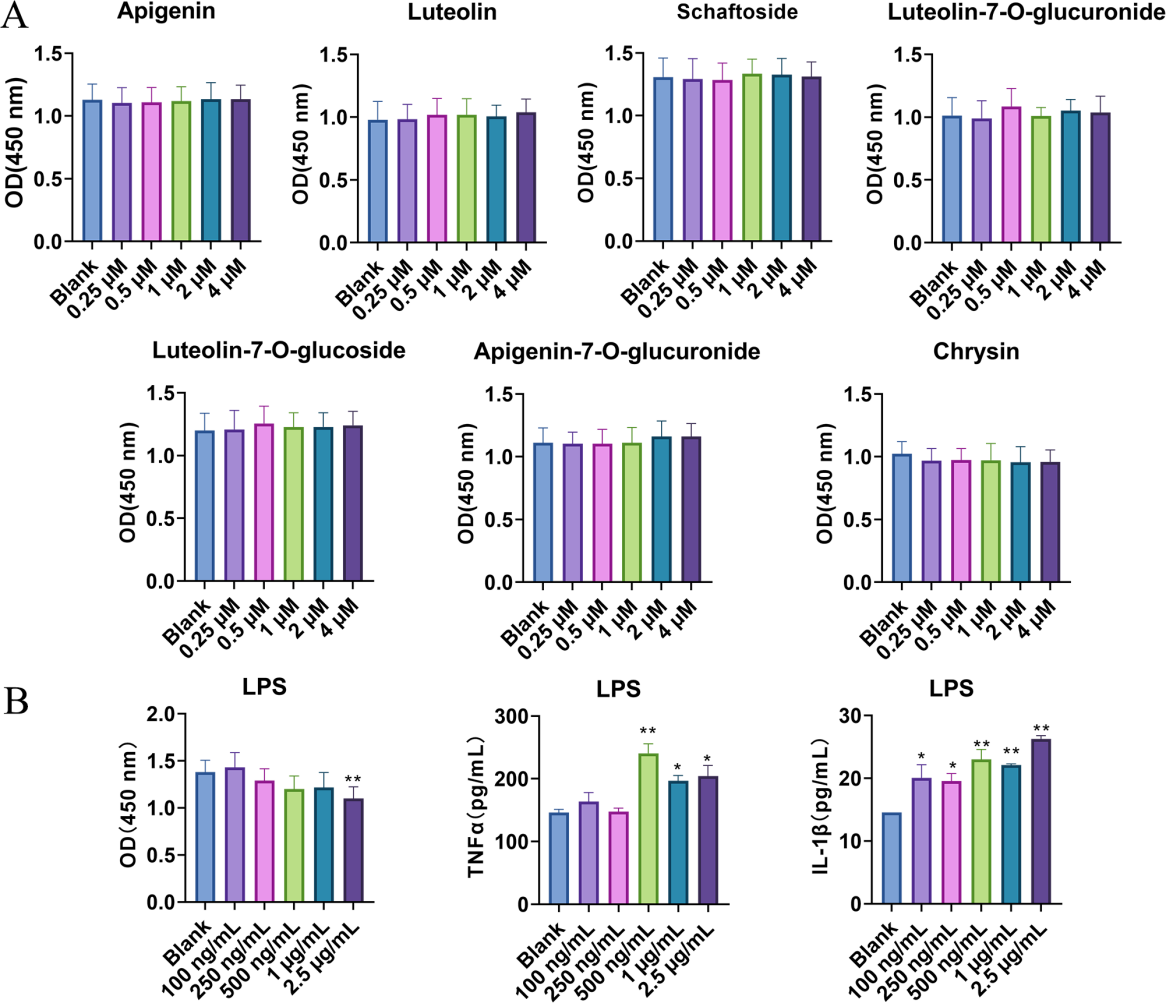
Effect of seven flavonoids on RAW264.7 cell viability and establishment of the LPS-induced inflammation model in RAW264.7 cells. **(A)** CCK-8 assay for cell viability. **(B)** The establishment of the LPS-induced RAW264.7 cell inflammation model. compared to the Blank group, *p < 0.05, **p < 0.01.

The cytotoxicity of seven flavonoids—apigenin, luteolin, schaftoside, luteolin-7-O-glucuronide, luteolin-7-O-glucoside, apigenin-7-O-glucuronide, and chrysin—was evaluated in RAW264.7 cells using the CCK-8 assay. None of these compounds exhibited significant cytotoxicity at concentrations up to 4 µM (**Figure 2A**).

To establish an appropriate inflammatory model, RAW264.7 cells were treated with varying concentrations of lipopolysaccharide (LPS). As shown in **Figure 2B**, LPS did not significantly affect cell viability at concentrations ≤1 µg/mL; however, higher concentrations markedly reduced viability (p < 0.01). Notably, treatment with 500 ng/mL LPS significantly elevated the secretion of TNF-α and IL-1β compared with the blank control (p < 0.01), without compromising cell viability. Therefore, 500 ng/mL LPS was selected for subsequent inflammation induction experiments.

**Figure 3:**
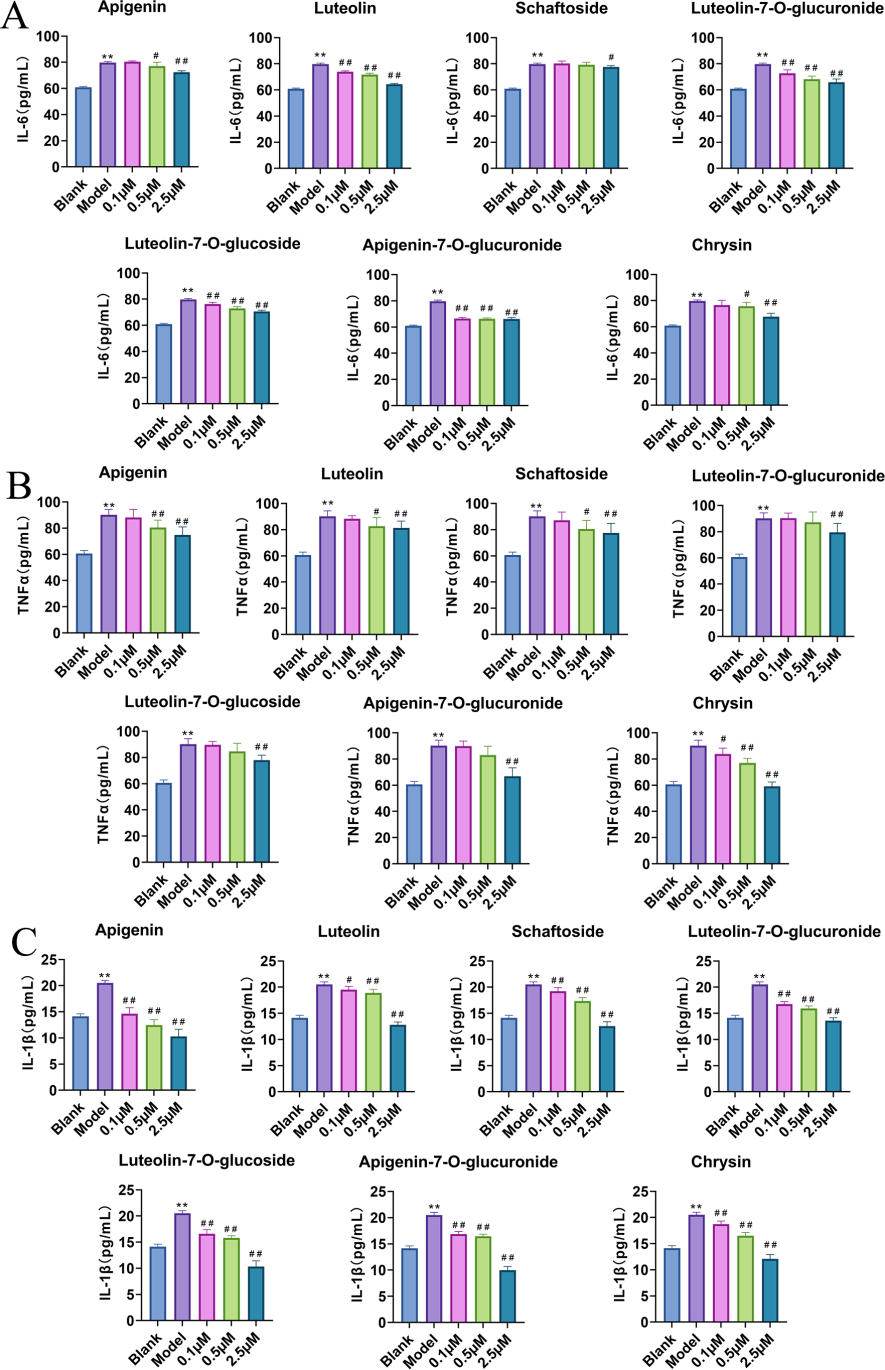
Effects of seven flavonoids on LPS-induced inflammation in RAW264.7 cells (n=6). **(A–C)** Concentration-dependent effects of apigenin, luteolin, schaftoside, luteolin-7-O-glucuronide, luteolin-7-O-glucoside, apigenin-7-O-glucuronide, and chrysin on the production levels of IL-6 **(A)**, TNF-α **(B)**, and IL-1β **(C)** in LPS-stimulated RAW264.7 cells. Compared to the Blank group, *p < 0.05, **p < 0.01, compared to the Model group, ^#^*p* < 0.05, ^##^*p* < 0.01.

The anti-inflammatory effects of the seven flavonoids were then assessed in the LPS-stimulated RAW264.7 model. Levels of IL-6, TNF-α, and IL-1β in cell supernatants were quantified by ELISA (**Figures 3A–C**). At a concentration of 2.5 µM, apigenin, luteolin, luteolin-7-O-glucuronide, luteolin-7-O-glucoside, apigenin-7-O-glucuronide, and chrysin all significantly suppressed the production of all three cytokines (p < 0.01). Schaftoside also markedly inhibited TNF-α and IL-1β (p < 0.01), though its effect on IL-6 was comparatively weaker. At a lower concentration (0.5 µM), apigenin, luteolin, and chrysin significantly reduced TNF-α levels (p < 0.05), whereas the glycosylated derivatives showed no significant effect. Furthermore, at 0.1 µM, apigenin, luteolin, and chrysin all significantly inhibited IL-1β production (p < 0.01), with apigenin demonstrating the most potent effect.

Collectively, these results indicate that all seven flavonoids can attenuate LPS-induced inflammatory responses in RAW264.7 cells by modulating the release of key pro-inflammatory cytokines, with apigenin and luteolin exhibiting potentially superior inflammatory activity.

### 3.2 Pharmacokinetic profile in mouse skin

**Figure 4:**
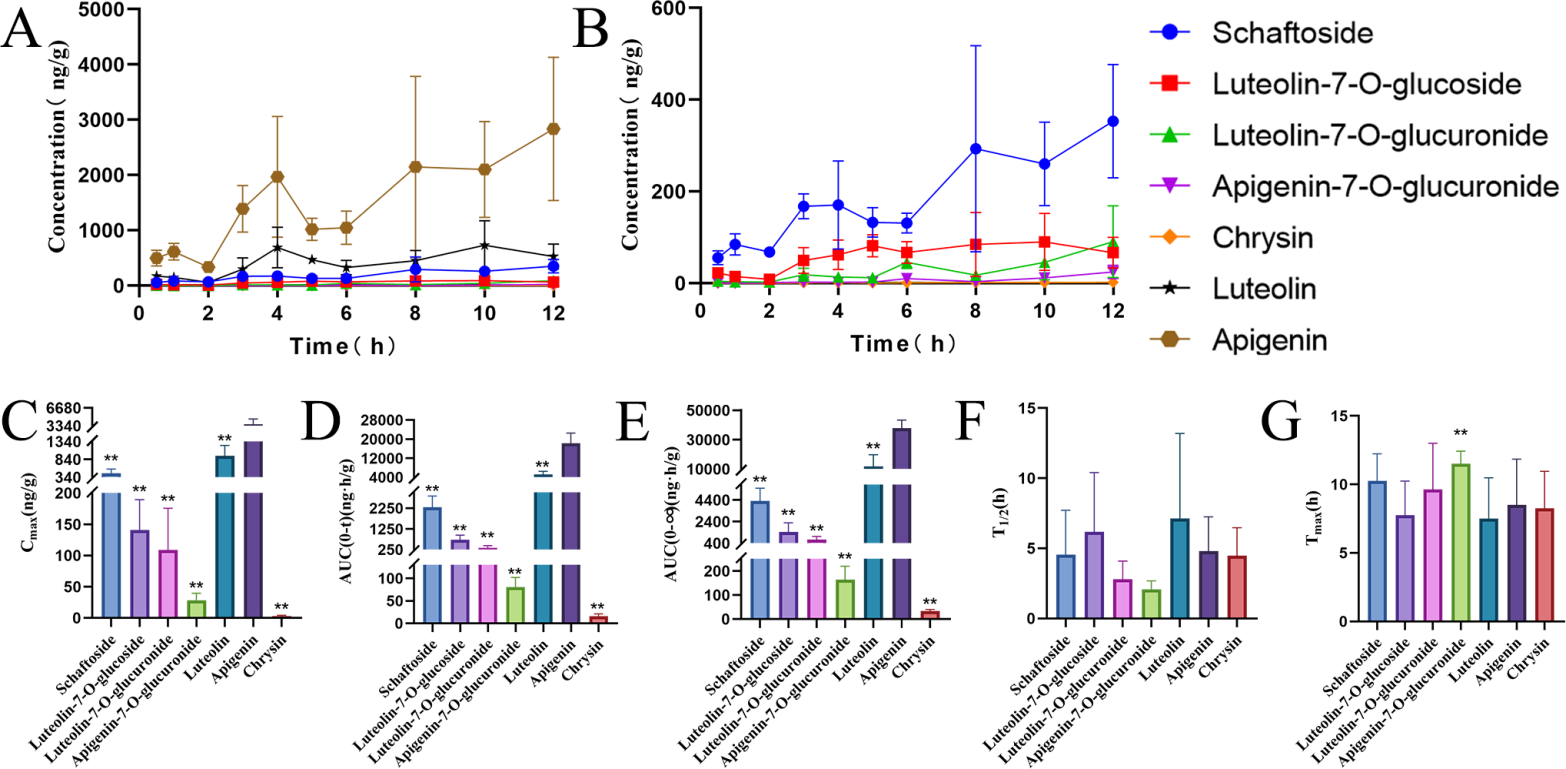
Skin pharmacokinetics of *Cayratia japonica* ointment (CJO)-derived flavonoids in mice. **(A)** Time–concentration profiles of seven flavonoids. **(B)** Detailed profiles of schaftoside, luteolin-7-O-glucuronide, luteolin-7-O-glucoside, apigenin-7-O-glucuronide, and chrysin. **(C–G)** Statistical comparisons of key PK parameters: C_max_, AUC_0–t_, AUC_0–∞_,T_1/2_, and T_max_. Data are presented relative to apigenin. *p < 0.05, **p < 0.01.

The transdermal pharmacokinetics of the seven flavonoids from CJO were evaluated in mouse skin (**Figures 4A–G**). Pharmacokinetic parameters were calculated using DAS 2.0 software (**Supplementary Table S7**). Among the tested compounds, apigenin exhibited the highest skin exposure, with significantly greater C_max_, AUC_0–t_, and AUC_0-∞_ values compared to the others flavonoids. Specifically, apigenin achieved a C_max_ of 3620.83 ± 1027.12 ng/g, which was significantly higher than that of luteolin (947.29 ± 285.90 ng/g, p < 0.01), schaftoside (449.17 ± 122.92 ng/g, *p* < 0.01), and all other identified flavonoids (*p* < 0.01). Apigenin’s exposure was further confirmed by AUC metrics (AUC_0–t_ = 18327.45 ± 4253.26 ng·h/g; AUC_0–∞_ = 37799.78 ± 5528.09 ng·h/g), which were 3.5- and 3.1-fold greater than those of luteolin, respectively (*p* < 0.01). Despite inter-animal variability, all CJO-treated mice maintained apigenin levels well above the *in vitro* effective concentration of 134.5 ng/mL (0.5 µM) required for significant inhibition of IL-6, TNF-α, and IL-1β. Given that the measured skin concentrations represent total tissue levels (ng/g), while *in vitro* activity is assessed in aqueous medium (ng/mL), direct quantitative comparison is limited. Nevertheless, the >26-fold higher magnitude of apigenin accumulation strongly suggests that therapeutically relevant exposure was achieved locally. Similarly, luteolin reached 947.29 ng/g—over 6-fold above its *in vitro* threshold (>0.5 µM, equivalent to ~143.12 ng/mL)—supporting its potential contribution to anti-inflammatory effects. In contrast, schaftoside attained only 449.17 ± 122.92 ng/g, which is below its minimal effective concentration (>2.5 µM, equivalent to ~ 1411.25 ng/mL) required for IL-6 suppression, indicating it is unlikely to play a major role in the local pharmacological activity of CJO.

At 12 h post-administration, cumulative absorption (AUC_0-t_/AUC_0-∞_) remained below 50% for all compounds except luteolin-7-O-glucoside, suggesting prolonged retention in skin tissue. The elimination half-life (T_1/2_) followed the order: apigenin-7-O-glucuronide > luteolin-7-O-glucuronide > chrysin > schaftoside > apigenin > luteolin > luteolin-7-O-glucoside. Notably, apigenin had a T_1/2_ of 4.81 ± 2.44 h, comparable to that of chrysin (4.48 ± 1.99 h) and schaftoside (4.55 ± 3.18 h) (non-significant, p> 0.05).

The time to peak concentration (T_max_) indicated the following absorption rate ranking: luteolin > luteolin-7-O-glucoside > chrysin > apigenin > luteolin-7-O-glucuronide > schaftoside > apigenin-7-O-glucuronide. However, the T_max_ of apigenin (8.50 ± 3.34 h) was not significantly different from those of luteolin (7.50 ± 2.98 h), luteolin-7-O-glucoside (7.75 ± 2.49 h), or chrysin (8.25 ± 2.71 h) (for detailed measurement processes, see *Appendix A*).

### 3.3 Apigenin promotes wound healing in a rat perianal abscess model

**Figure 5:**
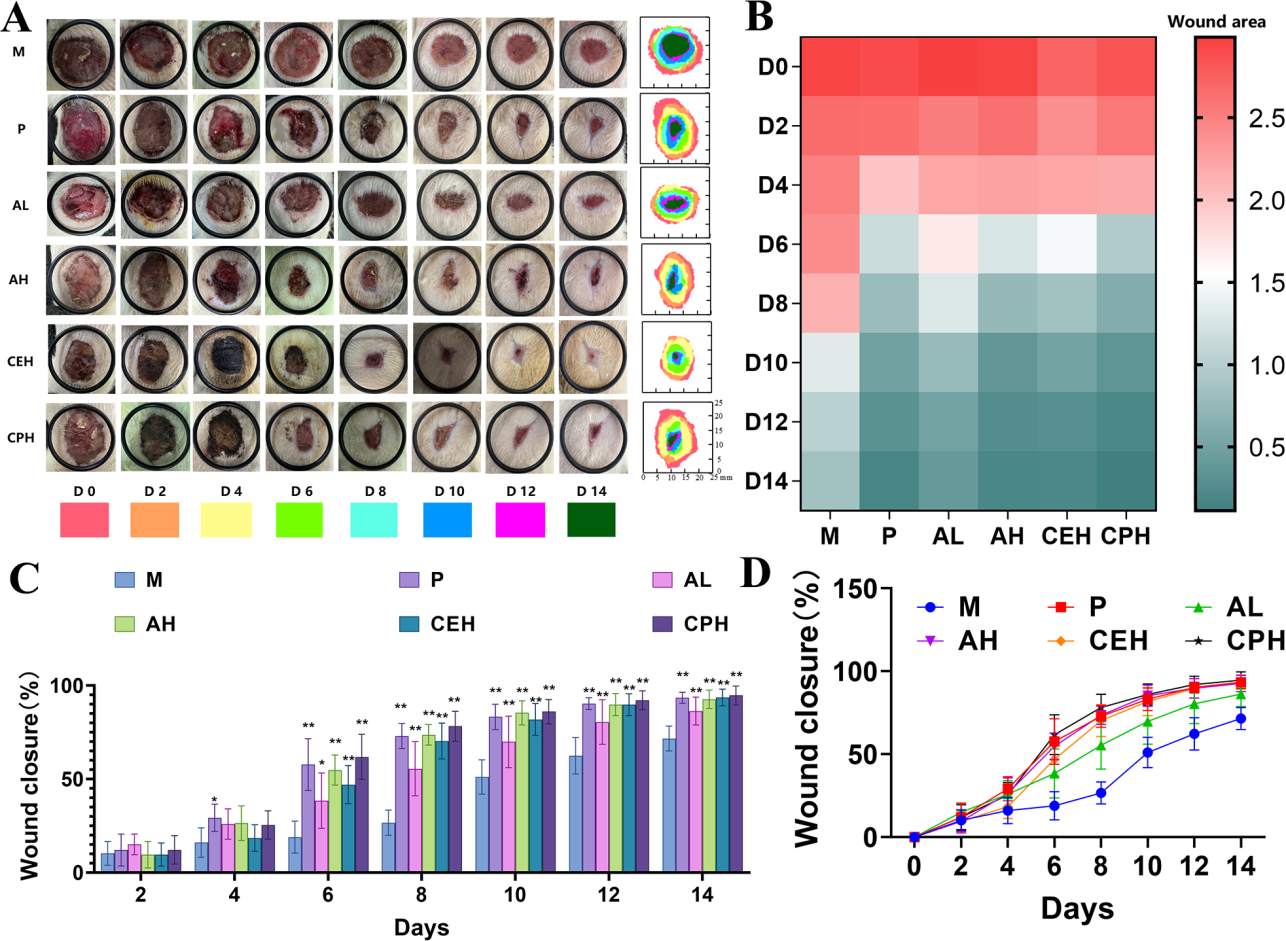
Postoperative wound healing in rats with perianal abscess across treatment groups. **(A)** Representative photographs and wound margin tracings from day 0 to day 14 for the Model (M), Positive control (P), Apigenin low-dose (AL), Apigenin high-dose (AH), *Cayratia japonica* extract (CEH), and *Cayratia japonica* powder (CPH) groups. **(B)** Wound area (cm²) over time. **(C, D)** Wound healing rates (%) at indicated time points (n=8). Compared to the Model group, *p < 0.05, **p < 0.01.

A postoperative perianal abscess model was established in rats to evaluate the wound-healing efficacy of apigenin. As shown in **Figures 5A–D**, apigenin treatment accelerated wound closure compared with the model (M) group. Persistent infection and inflammation were observed in the M group on days 6 and 8, whereas the apigenin-treated (AL, AH), positive control (P), and CJO-treated (CEH, CPH) groups showed minimal signs of infection.

Quantitative analysis revealed that by day 4, all treatment groups exhibited numerically faster wound closure than the M group, though these differences were not statistically significant (*p* > 0.05 by one-way ANOVA with Tukey’s *post-hoc* test), with only the P group showing a statistically significant improvement (*p* < 0.05).

From days 6 to 14, wound healing rates in all treated groups were significantly higher than in the M group (*p* < 0.05 or *p*< 0.01). The high-dose apigenin (AH) group healed significantly faster than the low-dose (AL) group (*p* < 0.05 or *p* < 0.01), while no significant differences were observed between AH and the CJO groups (CEH, CPH). These findings suggest that apigenin exerts its primary therapeutic effect during the inflammatory phase of wound healing and that its efficacy is comparable to that of the full CJO formulation.

**Figure 6:**
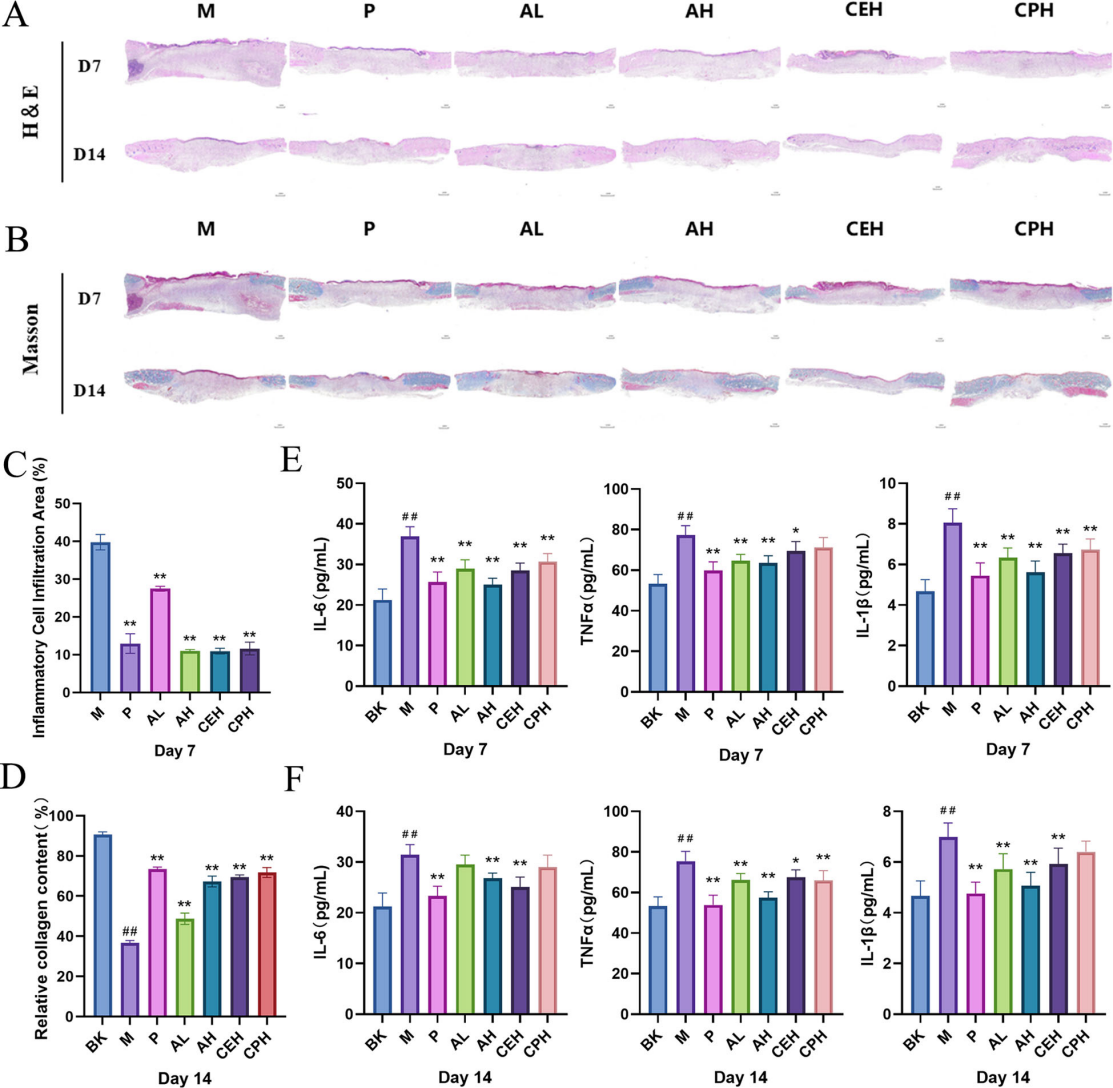
Histological evaluation and systemic inflammatory responses in rats with perianal abscess across treatment groups. **(A)** Representative H&E-stained wound tissue sections from the Model (M), Positive control (P), Apigenin low-dose (AL), Apigenin high-dose (AH), *Cayratia japonica* extract (CEH), and *Cayratia japonica* powder (CPH) groups on days 7 and 14 (scale bar: 1 mm). **(B)** Corresponding Masson’s trichrome–stained sections showing collagen deposition (scale bar: 1 mm). **(C)** The percentage of the wound area occupied by inflammatory cell infiltration on day 7 (n = 3 sections/animal, 5 fields/section). **(D)** The relative collagen content in the wound tissue on day 14 (n = 3 sections/animal, 5 fields/section). **(E, F)** Serum levels of IL-6, TNF-α, and IL-1β on day 7 **(E)** and day 14 **(F)** (n = 6 per group). Compared to the BK group, ^#^*p* < 0.05, ^##^*p* < 0.01, compared to the M group, *p < 0.05, **p < 0.01.

Histopathological evaluation further supported these findings. H&E staining on day 7 revealed persistent inflammatory cell infiltration in the M (model) group, whereas all treated groups exhibited significantly reduced inflammation compared to the M group (*p* < 0.01). Among the treatments, the AH group demonstrated the greatest attenuation of inflammatory infiltration. This improvement was significantly greater than that observed in the AL group (*p* < 0.01) and showed a numerical (though not statistically significant) advantage over the CEH and CPH groups (*p* > 0.05) (**Figures 6A, C**). Masson’s trichrome staining on day 14 showed significantly enhanced collagen deposition and improved wound architecture in all treated groups c relative to the M group (*p* < 0.01). Collagen accumulation in the AH group was significantly greater than in the AL group (p < 0.01) but marginally lower than in the CEH and CPH groups, though this difference did not reach statistical significance (*p* > 0.05) (**Figures 6B, D**). This modest enhancement may be attributed to additional bioactive constituents in CJO—such as luteolin—which have been reported to stimulate fibroblast proliferation and collagen synthesis, potentially acting synergistically with apigenin’s anti-inflammatory effects (29). Serum ELISA results demonstrated that all treatments suppressed systemic inflammation to varying degrees (**Figures 6E, F**).

On day 7, the AL, AH, and CEH groups significantly reduced IL-6, TNF-α, and IL-1β levels (p < 0.05). The CPH group suppressed IL-6 and IL-1β (*p* < 0.01) but not TNF-α. Notably, the AH group exhibited stronger inhibition of IL-6 and IL-1β than the AL group (*p* < 0.05), with no significant difference in TNF-α suppression between them. Moreover, the AH group demonstrated significantly greater efficacy than both CEH and CPH groups in suppressing all three cytokines (*p* < 0.05), indicating dose-dependent anti-inflammatory effects of apigenin that surpassed those of CJO.

By day 14, TNF-α was significantly suppressed in all treatment groups (*p* < 0.01). IL-6 and IL-1β were markedly reduced in the AH and CEH groups (*p* < 0.01), while only IL-1β was significantly lowered in the AL group; the CPH group showed no significant effect on either. The AH group demonstrated superior inhibition of IL-6 and TNF-α compared to AL (*p* < 0.05), and stronger suppression of TNF-α and IL-1β than CPH (*p* < 0.05).

Importantly, after 14 days, serum levels of TNF-α and IL-1β in the AH group were comparable to those in the blank (BK) group, with only IL-6 remaining significantly elevated (*p* < 0.01). In contrast, the AL, CEH, and CPH groups still exhibited markedly higher levels of all three cytokines than BK (*p* < 0.01). These findings suggest that high-dose apigenin nearly normalized systemic inflammatory cytokine levels, outperforming both low-dose apigenin and CJO formulations.

### 3.4 Apigenin modulates the JAK1/STAT3 signaling pathway

**Figure 7:**
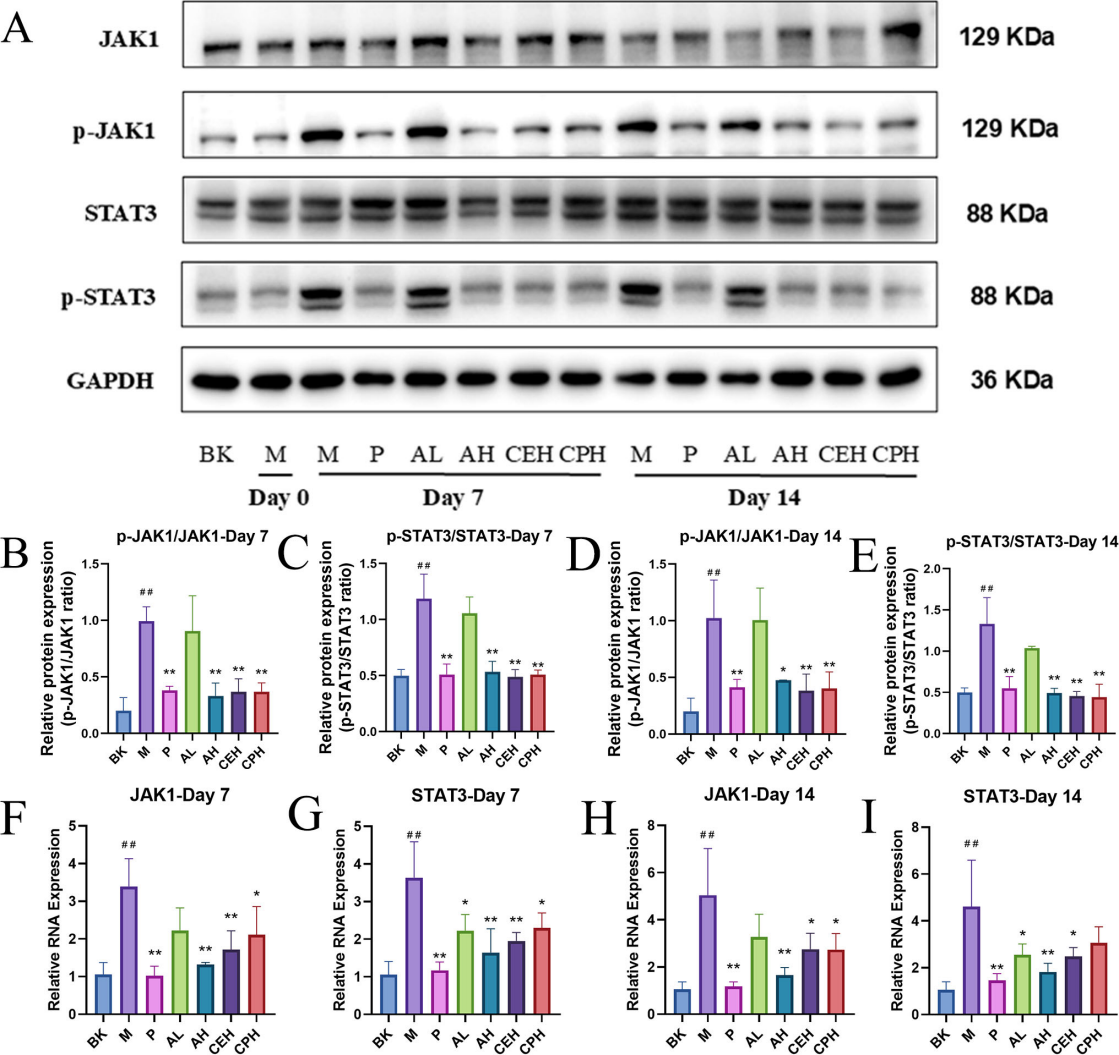
Expression of JAK1 and STAT3 in wound tissues of rats with perianal abscess following treatment. **(A–E)** Representative Western blot analysis of JAK1, phosphorylated JAK1 (p-JAK1), STAT3, and phosphorylated STAT3 (p-STAT3) in skin tissues from the Blank (BK), Model (M), Positive control (P), Apigenin low-dose (AL), Apigenin high-dose (AH), *Cayratia japonica* extract (CEH), and *Cayratia japonica* powder (CPH) groups at days 7 and 14. GAPDH served as the loading control (n = 3). **(F–I)** RT-qPCR analysis of JAK1 and STAT3 mRNA levels in the same tissue samples (n = 3). Compared to the BK group, ^#^*p* < 0.05, ^##^*p* < 0.01, compared to the M group, *p < 0.05, **p < 0.01. (Day 0 samples were collected immediately after wound creation and prior to both inflammatory challenge and drug administration; consequently, JAK1/STAT3 phosphorylation remains at basal levels due to the absence of sustained inflammatory stimuli).

To elucidate the molecular mechanism underlying apigenin’s effects, we examined its impact on the JAK1/STAT3 pathway (**Figures 7A–I**). Western blot analysis showed that, at both 7 and 14 days, the AH, CEH, and CPH groups exhibited significantly reduced phosphorylation of JAK1 and STAT3 (*p*< 0.01), with the AL group showing a similar but non-significant trend. The inhibitory effect was dose-dependent, with AH outperforming AL (*p* < 0.05 or *p*< 0.01) (**Figures 7A–E**). Full raw blots with molecular weight markers are provided in Supplementary materials B. The low phosphorylation levels of JAK1/STAT3 in day 0 samples (BK and M groups) reflect basal signaling activity in uninjured/untreated tissues. Variations in control group bands were within acceptable experimental limits (CV < 15%) and did not affect result interpretation.

RT-qPCR results corroborated these findings: after 7 days, JAK1 and STAT3 mRNA levels were significantly downregulated in the AH, CEH, and CPH groups (*p*< 0.05 or *p* < 0.01), with AH showing the strongest suppression. By day 14, JAK1 and STAT3 expression remained significantly lower in the AH and CEH groups compared with the M group (*p* < 0.05), and the AH group exhibited greater inhibition than both CEH and CPH (**Figures 7F–I**).

**Figure 8:**
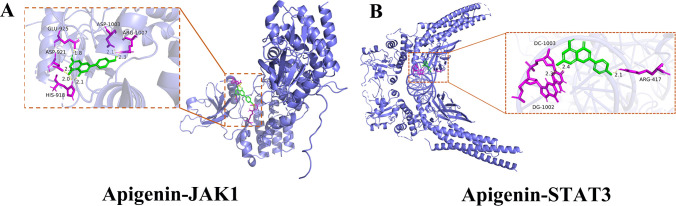
Molecular docking models illustrating the interactions between apigenin and JAK1/STAT3 pathway targets. **(A)** Apigenin docked into the binding pocket of JAK1. **(B)** Apigenin docked into the binding pocket of STAT3.

Molecular docking simulations revealed that apigenin forms stable hydrogen bonds with key residues in JAK1 (ARG-1007, ASP-921, ASP-1003, GLU-925) and STAT3 (ARG-609, SER-611, LY-S591), with binding energies of –8.2 and –8.4 kcal/mol, respectively, indicating strong affinity (**Figures 8A, B**). These data suggest that apigenin may suppress inflammatory responses via modulation of JAK1/STAT3 signaling.

These findings collectively demonstrate that apigenin may contribute to observed biological effects of CJO, promoting postoperative wound healing in rats by suppressing inflammation through potential modulation of the JAK1/STAT3 signaling pathway.

## 4 Discussion

Our study provides compelling evidence that apigenin, a major flavonoid constituent of CJO, is the principal driver of its therapeutic efficacy in postoperative perianal abscess wound healing. By integrating *in vitro* immunopharmacology, *in vivo* validation, skin pharmacokinetics, and molecular docking, we demonstrate that apigenin exerts potent anti-inflammatory effects primarily through potential modulation of the JAK1/STAT3 signaling pathway in macrophages—a mechanism highly relevant to the resolution of chronic wound inflammation.

The central role of macrophages in orchestrating the inflammatory phase of wound repair is well established (30). Excessive macrophage activation leads to sustained release of pro-inflammatory cytokines (TNF-α, IL-1β, IL-6), which disrupts the balance of the healing process and impairs tissue regeneration—effects that are closely linked to hyperactivation of the JAK/STAT pathway (31, 32). In our LPS-stimulated RAW264.7 model, apigenin emerged as the most effective among seven CJO-derived flavonoids in suppressing these key pro-inflammatory cytokines, even at low micromolar concentrations. This superior activity aligns with previous reports on apigenin’s immunomodulatory potential in colitis and dermatitis models (33, 34), yet our work is the first to link it to perianal wound healing. While RAW264.7 cells provided a practical and reproducible model for initial flavonoid screening, they do not fully recapitulate the heterogeneity of tissue-resident macrophages *in vivo*—including M1/M2 polarization states and tissue-specific functional adaptations. Future studies should therefore validate apigenin’s immunomodulatory effects in human skin macrophages isolated from wound edge biopsies, as well as in macrophages differentiated from peripheral blood monocytes under M1 (IFN-γ/LPS), M2 (IL-4/IL-13), and wound-relevant (TGF-β/PDGF) polarization conditions to assess context-dependent efficacy.

Critically, we established a pharmacokinetic–pharmacodynamic (PK–PD) correlation: apigenin achieved the highest skin exposure (C_max_ = 3620.83 ± 1027.12 ng/g), followed by luteolin (947.29 ± 285.90 ng/g) among all tested flavonoids following topical CJO application—both markedly exceeding the levels of other constituents. Quantitative analysis revealed that per gram of CJO (containing 150 mg *Cayratia japonica* powder), the contents of apigenin was 17.72 µg—lower than those of glycosylated flavonoids (e.g., apigenin-7-O-glucuronide: 317.19 µg). This confirms that apigenin not only is bioactive but also reaches therapeutically relevant levels at the target site, explaining why it, rather than more abundant but less potent analogs (e.g., luteolin-7-O- glucuronide), dominates CJO’s efficacy. While our PK/PD integration represents a significant advancement, complete bioavailability assessment would require measurement of unbound drug concentrations at the precise subcellular target site. Future studies using fluorescently labeled apigenin with super-resolution microscopy could provide nanoscale spatial resolution of target engagement.

In the rat perianal abscess model, high-dose apigenin (AH) significantly accelerated wound closure, reduced inflammatory cell infiltration, and enhanced collagen deposition—effects comparable to the full CJO formulation. This finding is of substantial translational significance: it suggests that a single, well-characterized compound can recapitulate the benefits of a complex herbal mixture, offering advantages in quality control, safety profiling, and regulatory approval. While apigenin matched CJO’s anti-inflammatory efficacy, the marginally superior collagen deposition in CJO-treated wounds highlights a critical principle in complex tissue repair: optimal healing requires sequential modulation of multiple pathways. CJO’s minor components (luteolin, schaftoside) likely activate complementary proliferative mechanisms during the repair phase, whereas isolated apigenin primarily targets the inflammatory phase. This pharmacodynamic complementarity supports the traditional ‘multi-target, multi-stage’ rationale for whole-herb formulations in wound management, suggesting reductionist approaches may compromise holistic tissue regeneration.

**Figure 9:**
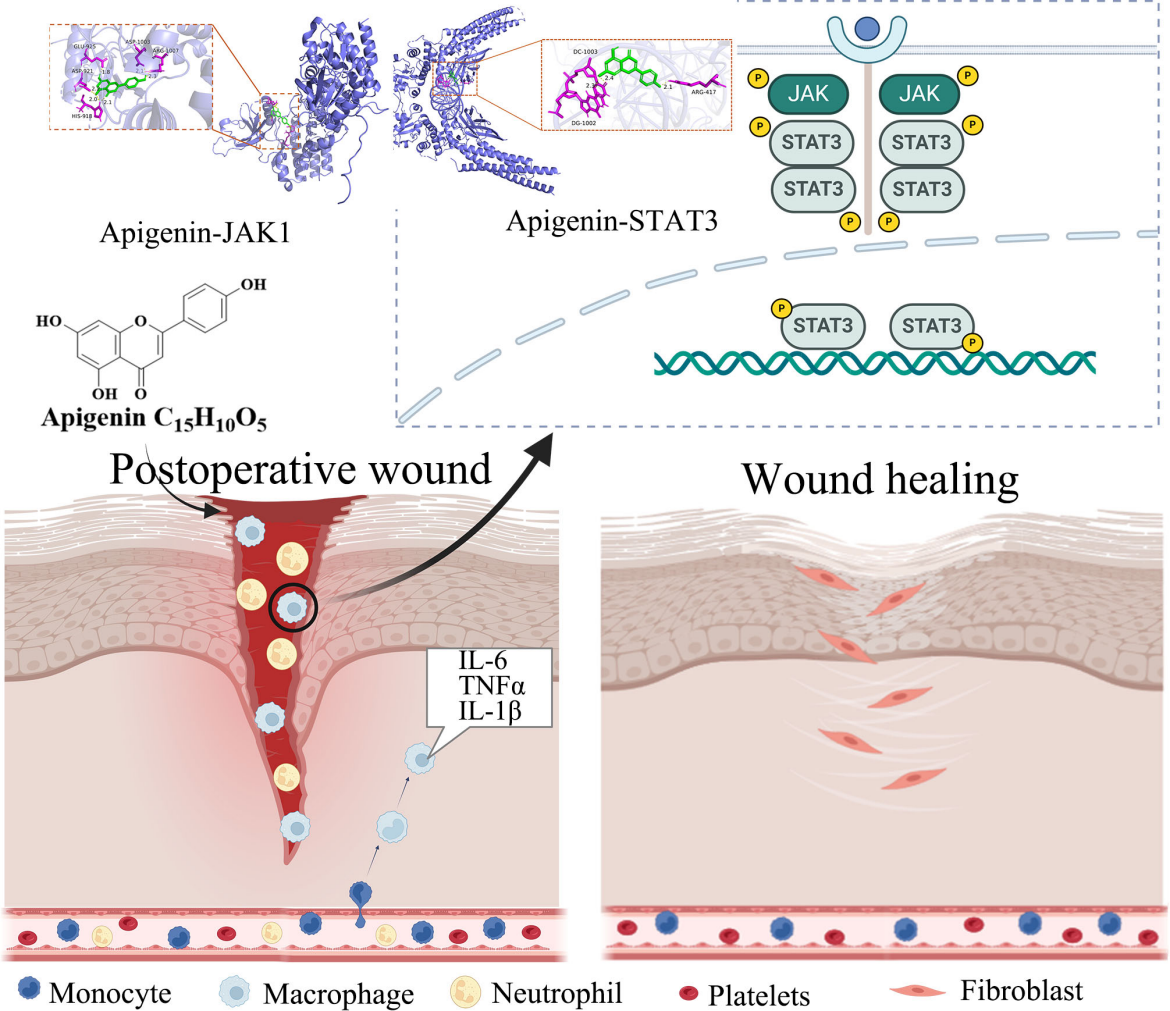
Apigenin exerts anti-inflammatory and pro-healing effects during wound repair via regulation of the JAK1/STAT3 pathway.

Mechanistically, we propose the JAK1/STAT3 axis as a potential molecular target of apigenin. Western blot and RT-qPCR revealed significant downregulation of JAK1 and STAT3 expression and phosphorylation in wound tissues after apigenin treatment. The observed reduction in *JAK1/STAT3* mRNA implies that apigenin’s effect extends beyond post-translational modification and involves broader anti-inflammatory transcriptional reprogramming. Molecular docking further confirmed stable hydrogen bonding between apigenin and key residues in JAK1 and STAT3, with binding energies (–8.2 and –8.4 kcal/mol) indicative of strong affinity. Consistently, Western blot and RT-qPCR analyses demonstrated that apigenin dose-dependently downregulated JAK1/STAT3 expression and phosphorylation in wound tissue (**Figure 9**), surpassing the effects of CJO. While the CEH and CPH groups showed similar reductions in p-JAK1/p-STAT3, CEH exhibited greater suppression of total JAK1/STAT3 mRNA, possibly reflecting compositional differences in CJO batches. However, future studies employing direct binding assays—such as surface plasmon resonance (SPR), isothermal titration calorimetry (ITC), or JAK1 kinase activity assays—are needed to confirm a direct physical interaction and establish binding affinity. Given that the JAK/STAT pathway is a master regulator of cytokine signaling in macrophages (35), its inhibition provides a plausible explanation for the broad suppression of TNF-α, IL-1β, and IL-6 observed *in vitro* and *in vivo*.

Notably, while other flavonoids like luteolin also inhibit JAK/STAT (36), their lower skin permeability or faster metabolism may limit their local efficacy—highlighting that dermal accumulation is as crucial as intrinsic potency in topical applications.

It is well established that LPS activates TLR4, leading to NF-κB–dependent upregulation of JAK1 and STAT3 in macrophages and microglia (37, 38). Apigenin has been shown to inhibit NF-κB nuclear translocation and modulate p38-MAPK signaling, thereby suppressing the expression of multiple inflammatory mediators—including upstream kinases and transcription factors such as JAK1 and STAT3 (39, 40). This suggests that apigenin attenuates LPS-induced JAK1/STAT3 signaling not only by reducing phosphorylation but also by inhibiting the transcriptional induction of JAK1 and STAT3 mRNA, likely through upstream suppression of NF-κB and MAPK pathways.

Several limitations warrant mention. First, we did not assess macrophage polarization markers (e.g., iNOS/Arg1) to directly confirm M1-to-M2 transition. Second, potential effects on other immune cells (e.g., neutrophils, T cells) in the wound bed remain unexplored. Future studies using macrophage-specific knockout models or single-cell RNA sequencing could further dissect apigenin’s immunomodulatory network. Although our model induces robust inflammation and impaired healing resembling complicated perianal sepsis, it lacks the defining histopathological feature of true anal fistulas—an epithelialized or granulation-lined tract connecting the anal canal to the perianal skin. Future studies employing seton-based or infection-induced fistula models would enhance the clinical translatability of our findings. While apigenin is detectable in CJO and exhibits robust anti-inflammatory and wound-healing activity when administered alone, its relative contribution to the overall efficacy of CJO—versus other co-occurring phytochemicals (e.g., luteolin, luteolin-7-O-glucuronide, luteolin-7-O-glucoside, apigenin-7-O-glucuronide)—remains undetermined. Future studies employing apigenin-depleted CJO preparations or comparative evaluation of CJO batches standardized to high versus low apigenin content will be essential to establish apigenin’s role as a key active ingredient. Our conclusions regarding direct target engagement rely on computational docking and indirect functional evidence. Definitive validation requires biophysical binding assays (e.g., SPR, ITC) and enzymatic characterization of JAK1 kinase modulation by apigenin.

In conclusion, our work positions apigenin as a naturally derived and mechanism-validated, mechanism-based immunomodulator for postoperative wound management. By targeting the JAK1/STAT3 pathway in macrophages, it resolves pathological inflammation while promoting tissue repair—offering a promising natural alternative to conventional anti-inflammatory agents with fewer systemic side effects.

## Data Availability

The original contributions presented in the study are publicly available. This data can be found here: https://doi.org/10.6084/m9.figshare.31033048.

## References

[1] McCurdy JD Weng R Parlow S Dawkins YM Brar G Oliveira L . Video capsule endoscopy can identify occult luminal crohn’s disease in patients with isolated perianal fistulas. J Crohns Colitis. (2023) 17:1624–30. doi: 10.1093/ecco-jcc/jjad078, PMID: 37101357

[2] Wang H Jiang HY Zhang YX Jin HY Fei BY Jiang JL . Mesenchymal stem cells transplantation for perianal fistulas: a systematic review and meta-analysis of clinical trials. Stem Cell Res Ther. (2023) 14:103. doi: 10.1186/s13287-023-03331-6, PMID: 37101285 PMC10134595

[3] Sahnan K Adegbola SO Tozer PJ Watfah J Phillips RK . Perianal abscess. Bmj. (2017) 356:j475. doi: 10.1136/bmj.j475, PMID: 28223268

[4] Qi W Wang L Xu J Gao J . The efficacy and safety of Chinese herbal medicine for reducing wound complications after anal fistula surgery: A protocol for systematic review and meta-analysis. Med (Baltimore). (2022) 101:e32021. doi: 10.1097/MD.0000000000032021, PMID: 36482572 PMC9726318

[5] Zhao F Li Y Hu Q Xu J Zhang N Chen Y . Hydrogen sulfide as a therapeutic agent for diabetic wounds: effects on inflammation and fibroblast pyroptosis. Front Immunol. (2025) 16:1558443. doi: 10.3389/fimmu.2025.1558443, PMID: 40936902 PMC12420200

[6] Joshi H Anaya E Addanki A Almgren-Bell A Todd EM Morley SC . Mechanosensitivity of macrophage polarization: comparing small molecule leukadherin-1 to substrate stiffness. Front Immunol. (2025) 16:1420325. doi: 10.3389/fimmu.2025.1420325, PMID: 40114914 PMC11922956

[7] Peña OA Martin P . Cellular and molecular mechanisms of skin wound healing. Nat Rev Mol Cell Biol. (2024) 25:599–616. doi: 10.1038/s41580-024-00715-1, PMID: 38528155

[8] Sun X Liang Y Wang Y Sun C Wang X . Bisdemethoxycurcumin, a curcumin derivative, ameliorates adjuvant-induced arthritis by suppressing inflammatory reactions and macrophage migration. Chem Biol Interact. (2024) 387:110822. doi: 10.1016/j.cbi.2023.110822, PMID: 38056805

[9] Yang G Yang J Diao Z Long J Shu Z Liu C . Cascade-targeting pH/ROS microneedles promote scarless diabetic wound healing by macrophage metaboimmune reprogramming. Sci Adv. (2025) 11:eadw3834. doi: 10.1126/sciadv.adw3834, PMID: 41171927 PMC12577711

[10] Mangum KD denDekker A Li Q Tsoi LC Joshi AD Melvin WJ . The STAT3/SETDB2 axis dictates NF-κB-mediated inflammation in macrophages during wound repair. JCI Insight. (2024) 9:e179017. doi: 10.1172/jci.insight.179017, PMID: 39435663 PMC11530128

[11] de Lima-Vasconcellos TH Bovi Dos Santos G Móvio MI Donnici GK Badin GM de Araujo DR . Neuroprotection provided by polyphenols and flavonoids in photoreceptor degenerative diseases. Neural Regener Res. (2026) 21:908–22. doi: 10.4103/NRR.NRR-D-24-01638, PMID: 40364630 PMC12296498

[12] Sun J Zhao P Ding X Li F Jiang J Huang H . Cayratia japonica Prevents Ulcerative Colitis by Promoting M2 Macrophage Polarization through Blocking the TLR4/MAPK/NF-κB Pathway. Mediators Inflamm. (2022) 2022:1108569. doi: 10.1155/2022/1108569, PMID: 36619207 PMC9822765

[13] Su L Zheng X . Clinical observation on Cayratia Japonica Cream in treating anorectal abscess with fire-toxin amassment pattern. China J Traditional Chin Med Pharmacy. (2017) 32:3822–4.

[14] Zhao X Dai R Wang J Cao L Chen P Yao W . Analysis of the permeable and retainable components of Cayratia japonica ointment through intact or broken skin after topical application by UPLC-Q-TOF-MS/MS combined with *in vitro* transdermal assay. J Pharm BioMed Anal. (2024) 238:115853. doi: 10.1016/j.jpba.2023.115853, PMID: 37976992

[15] Waheed A Zameer S Ashrafi K Ali A Sultana N Aqil M . Insights into Pharmacological Potential of Apigenin through Various Pathways on a Nanoplatform in Multitude of Diseases. Curr Pharm Des. (2023) 29:1326–40. doi: 10.2174/1381612829666230529164321, PMID: 37254541

[16] Abid R Ghazanfar S Farid A Sulaman SM Idrees M Amen RA . Pharmacological properties of 4’, 5, 7-trihydroxyflavone (Apigenin) and its impact on cell signaling pathways. Molecules. (2022) 27:4304. doi: 10.3390/molecules27134304, PMID: 35807549 PMC9267958

[17] Demirci Yildirim T Kahraman A Köken Avşar A Onen F Akar S Sari İ . Quantitative analysis of JAK/STAT signaling pathway in patients of inflammatory skin disorders. Rheumatol Int. (2024) 44:3009–15. doi: 10.1007/s00296-023-05418-y, PMID: 37558928

[18] Lin Z He H Chen P Dong X Jiang J Huang W . Alpha-ketoglutarate protects against myocardial infarction via FTO-mediated anti-inflammatory macrophage activation. Basic Res Cardiol. (2025) 120:889–912. doi: 10.1007/s00395-025-01135-8, PMID: 40817969

[19] Yan Y Zhang LB Ma R Wang MN He J Wang PP . Jolkinolide B ameliorates rheumatoid arthritis by regulating the JAK2/STAT3 signaling pathway. Phytomedicine. (2024) 124:155311. doi: 10.1016/j.phymed.2023.155311, PMID: 38199156

[20] Li Q Xiang Y Zhang Z Qu X Wu J Fu J . An integrated RNA-Seq and network pharmacology approach for exploring the preventive effect of Corydalis bungeana Turcz. Extract and Acetylcorynoline on LPS-induced acute lung injury. J Ethnopharmacol. (2024) 318:117048. doi: 10.1016/j.jep.2023.117048, PMID: 37586441

[21] Lu YT Guan R Zheng JR Hang TJ Song M . Determination of clobetasol propionate, tazarotene and tazarotenic acid in Bama mini-pig skin by UPLC-MS/MS: Application to pharmacokinetic and drug-drug interaction studies. J Pharm BioMed Anal. (2020) 186:113322. doi: 10.1016/j.jpba.2020.113322, PMID: 32380355

[22] Zhang J Si H Sun J Lv K Yan B Li B . Determination of myrislignan levels in BALB/c mouse plasma by LC-MS/MS and a comparison of its pharmacokinetics after oral and intraperitoneal administration. BMC Vet Res. (2021) 17:275. doi: 10.1186/s12917-021-02990-y, PMID: 34399756 PMC8365968

[23] Wang L Qi W Gao J Tian M Xu J . Tongyangxiao Lotion promotes postoperative wound healing in a rat model of anal fistula by downregulating inflammatory factors and suppressing inflammation. J South Med University. (2024) 44:1256–65. doi: 10.12122/j.issn.1673-4254.2024.07.05, PMID: 39051071 PMC11270659

[24] Guidance for industry FDA . bioanalytical method validation. U.S. Dep. Heal.Hum. Serv. (2018) p:1–44.

[25] Rajoo A Ramanathan S Mansor SM Sasidharan S . Formulation and evaluation of wound healing activity of Elaeis guineensis Jacq leaves in a Staphylococcus aureus infected Sprague Dawley rat model. J Ethnopharmacol. (2021) 266:113414. doi: 10.1016/j.jep.2020.113414, PMID: 32980488

[26] Vasanthi V Divya B Ramadoss R Deena P Annasamy RK Rajkumar K . Quantification of inflammatory, angiogenic, and fibrous components of reactive oral lesions with an insight into the pathogenesis. J Oral Maxillofac Pathol. (2022) 26:600. doi: 10.4103/jomfp.jomfp_138_21, PMID: 37082049 PMC10112086

[27] Ramadoss R Krishnan R Vasanthi V Bose D Vijayalakshmi R Padmanabhan R . New insights for consummate diagnosis and management of oral submucous fibrosis using reactive and reparative fibrotic parameter derived algorithm. J Pharm Bioallied Sci. (2021) 13:S323–32. doi: 10.4103/jpbs.JPBS_822_20, PMID: 34447103 PMC8375801

[28] Chen K Li J Chen Z Shen C Li X Li Y . Notoginsenoside R1 alleviates blue light-induced corneal injury and wound healing delay by binding to and inhibiting TRIB1. Phytomedicine. (2025) 138:156399. doi: 10.1016/j.phymed.2025.156399, PMID: 39884079

[29] Wang T Pan D Zhang Y Li D Zhang Y Xu T . Luteolin antagonizes angiotensin II-dependent proliferation and collagen synthesis of cultured rat cardiac fibroblasts. Curr Pharm Biotechnol. (2015) 16:430–9. doi: 10.2174/1389201015666141110142402, PMID: 25382303

[30] Pomeyie K Abrokwah F Boison D Amoani B Kyei F Adinortey CA . Macrophage immunometabolism dysregulation and inflammatory disorders. BioMed Pharmacother. (2025) 188:118142. doi: doi: 10.1016/j.biopha.2025.118142, PMID: 40378771 10.1016/j.biopha.2025.118142

[31] Feng W Yang K Zou Y Xiao Z Qian R Qian R . Progress of ursolic acid on the regulation of macrophage: summary and prospect. Front Immunol. (2025) 16:1576771. doi: 10.3389/fimmu.2025.1576771, PMID: 40421013 PMC12104263

[32] Ghamangiz S Jafari A Maleki-Kakelar H Azimi H Mazloomi E . Reprogram to heal: Macrophage phenotypes as living therapeutics. Life Sci. (2025) 371:123601. doi: 10.1016/j.lfs.2025.123601, PMID: 40189197

[33] Huang Y Wang N Ji X Luo S Gong L Zhao C . Apigenin ameliorates inflamed ulcerative colitis by regulating mast cell degranulation via the PAMP-MRGPRX2 feedback loop. Phytomedicine. (2025) 140:156564. doi: 10.1016/j.phymed.2025.156564, PMID: 40054174

[34] Sung YY Kim M Kim DS Son E . Glycine soja leaf and stem extract ameliorates atopic dermatitis-like skin inflammation by inhibiting JAK/STAT signaling. Int J Mol Sci. (2025) 26:4560. doi: 10.3390/ijms26104560, PMID: 40429704 PMC12110808

[35] Zhao H Zhou Y Xu J Zhang Y Wang H Zhao C . Short-chain fatty acid-producing bacterial strains attenuate experimental ulcerative colitis by promoting M2 macrophage polarization via JAK/STAT3/FOXO3 axis inactivation. J Transl Med. (2024) 22:369. doi: 10.1186/s12967-024-05122-w, PMID: 38637862 PMC11025230

[36] Caporali S De Stefano A Calabrese C Giovannelli A Pieri M Savini I . Anti-inflammatory and active biological properties of the plant-derived bioactive compounds luteolin and luteolin 7-glucoside. Nutrients. (2022) 14:1155. doi: 10.3390/nu14061155, PMID: 35334812 PMC8949538

[37] Yu P Li Y Wang Y Peppelenbosch MP Pan Q . Lipopolysaccharide restricts murine norovirus infection in macrophages mainly through NF-kB and JAK-STAT signaling pathway. Virology. (2020) 546:109–21. doi: 10.1016/j.virol.2020.04.010, PMID: 32452409

[38] Ahuja A Kim E Sung GH Cho JY . STAT3 differentially regulates TLR4-mediated inflammatory responses in early or late phases. Int J Mol Sci. (2020) 21:7675. doi: 10.3390/ijms21207675, PMID: 33081347 PMC7589049

[39] Ai XY Qin Y Liu HJ Cui ZH Li M Yang JH . Apigenin inhibits colonic inflammation and tumorigenesis by suppressing STAT3-NF-κB signaling. Oncotarget. (2017) 8:100216–26. doi: 10.18632/oncotarget.22145, PMID: 29245972 PMC5725014

[40] Kim A Lee CS . Apigenin reduces the Toll-like receptor-4-dependent activation of NF-κB by suppressing the Akt, mTOR, JNK, and p38-MAPK. Naunyn Schmiedebergs Arch Pharmacol. (2018) 391:271–83. doi: 10.1007/s00210-017-1454-4, PMID: 29264665

