# Supplementary material for: Apigenin ameliorates postoperative inflammation and accelerates wound healing in a rat model of perianal abscess by targeting the JAK1/STAT3 pathway in macrophages
Source: Front Immunol. 2026 Jan 28;17:1726917. doi: 10.3389/fimmu.2026.1726917 (PMC12890627; doi:10.3389/fimmu.2026.1726917)
Supplement: Supplementary file 1 [file DataSheet1.docx]

Supplementary Material

**Apigenin, the transdermal ingredient in *Cayratia japonica* ointment, promotes wound healing after perianal abscess surgery by regulating JAK1/STAT3 pathway**

**Ruixue Dai *et al.***

# **Reagents and materials**

*Cayratia japonica* (Batch No. 221101) was obtained from Anhui Huchuntang Traditional Chinese Medicine Tea Slice Co., Ltd (Anhui, China). Vaseline Yellow (Batch No. A2220185) was purchased from Shanghai Aladdin Biochemical Technology Co., Ltd (Shanghai, China). Reference standards—including: esculetin (Purity: HPLC ≥ 98%; Batch No. C25J7Y18390), schaftoside (Purity: HPLC ≥ 98%; Batch No. D16GB171625), luteolin-7-O-glucuronide (Purity: HPLC ≥ 98%; Batch No. M31IB211439), luteolin-7-O-glucoside (Purity: HPLC ≥ 98%; Batch No. A22GB141264), apigenin-7-O-glucuronide (Purity: HPLC ≥ 98%; Batch No. Z18A7Z13273), luteolin (Purity: HPLC ≥ 98%; Batch No. S08GB160295), apigenin (Purity: HPLC ≥ 98%; Batch No. M29GB150104), and chrysin (Purity: HPLC ≥ 98%; Batch No. X24O6C4947) were purchased from Shanghai Yuanye Biotechnology Co., Ltd (Shanghai, China). Digoxin (Purity: HPLC ≥ 98%; Batch No. DST210420-086) was purchased from Chengdu Desite Biotechnology Co., Ltd (Chengdu, China). MS-grade methanol (Batch No. I1240335238) and acetonitrile (Batch No. I1223729233) were purchased from Merck KGaA (Darmstadt, Germany). Formic acid (MS grade, Batch No. 220622) was supplied by Thermo Fisher Scientific (Waltham, MA, USA).

# **Stock solutions, standards samples and quality control samples**

Accurately weighed reference standards of esculetin, schaftoside, luteolin-7-O-glucuronide, luteolin-7-O-glucoside, apigenin-7-O-glucuronide, luteolin, apigenin, chrysin, and digoxin were individually dissolved in 10 mL of methanol to prepare stock solutions with concentrations of 1.004, 1.000, 1.005, 1.000, 1.002, 1.003, 1.004, 1.002, and 1.000 mg/mL, respectively.

An equal-volume mixture of the eight analyte stock solutions (excluding digoxin) was prepared to generate a mixed standard solution. This mixture was serially diluted with methanol to yield working solutions for calibration standards (CS) and quality control (QC) samples at concentrations of 5000, 4000, 2000, 1000, 500, 400, 200, 100, 50, 40, 20, 10, 5, 4, 2, and 1 ng/mL. Additionally, two mixed working solutions were prepared for method validation:

Lower limit of quantification (LLOQ): 40.16, 5.000, 4.020, 4.000, 5.010, 10.03, 2.008, and 2.004 ng/mL for esculetin, schaftoside, luteolin-7-O-glucuronide, luteolin-7-O-glucoside, apigenin-7-O-glucuronide, luteolin, apigenin, and chrysin, respectively.

Low-concentration QC (QCL): 100.4, 10.00, 10.05, 10.00, 10.02, 20.06, 5.020, and 5.010 ng/mL for the same analytes.

Medium (QCM) and high (QCH) QC concentrations were set at 2000 and 4000 ng/mL, respectively, with the upper limit of quantification (ULOQ) at 5000 ng/mL.

# **Preparation of calibration standards (CS) and quality control (QC) samples**

A 0.1 g blank skin sample was homogenized with ultrapure water at a 1:9 (w/w) ratio to yield a tissue homogenate. To 600 µL of blank rat skin homogenate, 10 µL of digoxin (internal standard, IS) solution and 10 µL of CS or QC working solution were added. The mixture was vortexed for 3 min, followed by protein precipitation with 2400 µL of acetonitrile. After an additional 3 min of vortexing, samples were centrifuged at 13,000 rpm for 10 min. The supernatant was evaporated to dryness under vacuum and reconstituted in 100 µL of 50% aqueous acetonitrile (Watson water: acetonitrile = 1:1, v/v). A 2-µL aliquot of the reconstituted solution was injected for UPLC–MS/MS analysis.

# **Samples preparation**

A 0.1 g skin sample was homogenized with ultrapure water at a 1:9 (w/w) ratio to yield a tissue homogenate. To 600 µL of rat skin homogenate, 10 µL of digoxin (internal standard, IS) solution were added. The mixture was vortexed for 3 min, followed by protein precipitation with 2400 µL of acetonitrile. After an additional 3 min of vortexing, samples were centrifuged at 13,000 rpm for 10 min. The supernatant was evaporated to dryness under vacuum and reconstituted in 100 µL of 50% aqueous acetonitrile (Watson water: acetonitrile = 1:1, v/v). A 2-µL aliquot of the reconstituted solution was injected for UPLC–MS/MS analysis.

# **HPLC-MS/MS analysis Conditions**

The mobile phase consisted of (A) 0.1% (v/v) formic acid in water and (B) acetonitrile. Gradient elution was performed as follows: 5–17% B (0–0.5 min), 17% B (0.5–2.4 min), 17–40% B (2.4–2.5 min), 40–95% B (2.5–4.5 min), 95% B (4.5–6.5 min), 95–5% B (6.5–9 min), and 5% B (9–12 min). The flow rate was 0.4 mL/min, and the injection volume was 2 µL.

Mass spectrometry was operated in negative ion mode with the following parameters: nebulizer gas (GS1) 55 psi, heater gas (GS2) 55 psi, curtain gas (CUR) 30 psi, ion spray voltage −4500 V, and source temperature 550 °C. Precursor (Q1) and product (Q3) ions, along with optimized declustering potential (DP) and collision energy (CE), are listed in Table S1.

**TABLE S1** The mass spectral information of 8 chemical components of *Cayratia japonica* and the internal standard (digoxin).

| Analyte | Q1（*m/z*) | Q3（*m/z*) | DP（-eV） | CE（-eV） |
| --- | --- | --- | --- | --- |
| Esculetin | 176.9 | 132.9 | 50 | 24 |
| Schaftoside | 563.2 | 353.1 | 60 | 51 |
| Luteolin-7-O-glucoside | 447.2 | 285.0 | 94 | 38 |
| Luteolin-7-O-glucuronide | 461.1 | 285.0 | 62 | 37 |
| Apigenin-7-O-glucuronide | 445.2 | 269.1 | 40 | 35 |
| Luteolin | 285.1 | 150.9 | 80 | 34 |
| Apigenin | 269.1 | 151.0 | 59 | 33 |
| Chrysin | 253.2 | 62.9 | 60 | 51 |
| Digoxin | 779.4 | 649.2 | 100 | 50 |

## **Method Validation**

The bioanalytical method was validated in accordance with FDA (2018) guidelines for bioanalytical method validation, including assessments of selectivity, carryover, linearity, sensitivity (LOD/LLOQ), precision, accuracy, matrix effect, recovery, dilution integrity, and sample stability.

## **Selectivity and Carryover**

To assess selectivity, blank rat skin homogenate was spiked with a mixture of the eight analytes—esculetin, schaftoside, luteolin-7-O-glucuronide, luteolin-7-O-glucoside, apigenin-7-O-glucuronide, luteolin, apigenin, and chrysin—at a nominal concentration of 50 ng/mL to generate a standard skin sample. The following sample types were analyzed by HPLC–MS/MS: Blank skin homogenate, Blank skin homogenate spiked with internal standard (IS, digoxin, 100 µg/mL), Blank skin homogenate spiked with analytes (50 ng/mL) and IS, Skin samples collected 12 h after topical application of *Cayratia japonica* ointment (CJO), and Blank solvent (matrix-free).

For carryover assessment, a mixed standard sample at the upper limit of quantification (ULOQ, 5000 ng/mL for each analyte) containing IS (100 µg/mL) was injected, immediately followed by an injection of blank solvent. Carryover was considered acceptable if the peak areas of all analytes and the IS in the blank solvent injection were ≤20% and ≤5%, respectively, of the peak area observed at the lower limit of quantification (LLOQ).

Representative extracted ion chromatograms (XICs) for the above sample types are shown in Figure S1A–E. No significant interference from endogenous skin components or residual compounds from the analytical system was observed at the retention times of the analytes or the internal standard, confirming adequate selectivity and negligible carryover.

**
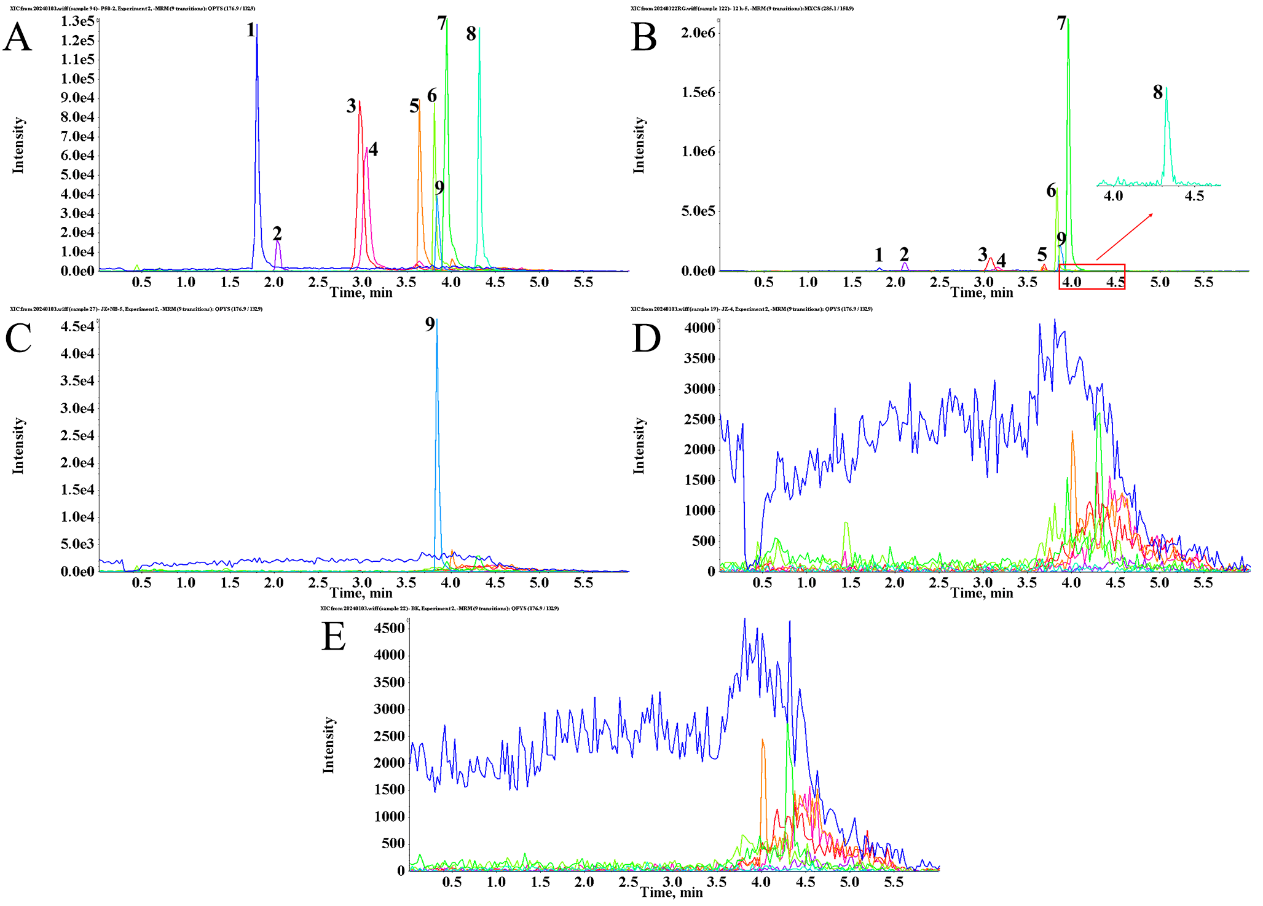
FIGURE S1**

Extracted ion chromatograms (XICs) of the eight target analytes and the internal standard (IS) from *Cayratia japonica*. (**A**) Blank rat skin homogenate spiked with mixed reference standards and IS. (**B**) Skin tissue collected 12 h after topical application of CJO. (**C**) Blank skin homogenate spiked with IS only. (**D**) Untreated blank skin homogenate. (**E**) Blank solvent. (Peak assignments: 1: esculetin; 2: schaftoside; 3: luteolin-7-O-glucuronide; 4: luteolin-7-O-glucoside; 5: apigenin-7-O-glucuronide; 6: luteolin; 7: apigenin; 8: chrysin; 9: digoxin)

## **Sensitivity and Linearity**

Calibration standards (CS) were prepared in triplicate at concentrations of 500, 400, 200, 100, 50, 40, 20, 10, 5, 4, 2, 1, 0.5, 0.4, 0.2, and 0.1 ng/mL for each analyte. Calibration curves were constructed by plotting the peak area ratio of the analyte to the internal standard (y) against the corresponding nominal concentration (x). A weighted (1/x²) least-squares linear regression model was applied to generate the calibration equations for all eight compounds.

As summarized in Table S2, all eight analytes exhibited excellent linearity over their respective concentration ranges, with correlation coefficients (r) ranging from 0.9976 to 0.9998, confirming the reliability and sensitivity of the developed HPLC–MS/MS method for quantitative analysis in skin tissue.

**TABLE S2** Limit of detection (LOD), lower limit of quantification (LLOQ), and calibration curve linearity for the eight target analytes in rat skin homogenate (n = 3).

| No. | Analyte | Linearity range /(ng/mL) | Linear equation | R | LOD (ng/mL) | LLOD  (ng/mL) |
| --- | --- | --- | --- | --- | --- | --- |
| 1 | Esculetin | 4.02~502 | y=0.02x+0.0803 | 0.9976 | 4.02 | 10.0 |
| 2 | Schaftoside | 0.500~500 | y=0.00359x+0.00161 | 0.9998 | 0.500 | 1.00 |
| 3 | Luteolin-7-O-glucuronide | 0.402~503 | y=0.0115x+0.000637 | 0.9996 | 0.402 | 1.01 |
| 4 | Luteolin-7-O-glucoside | 0.400~500 | y=0.0225x+0.00183 | 0.9995 | 0.400 | 1.00 |
| 5 | Apigenin-7-O-glucuronide | 0.501~501 | y=0.00465x+0.00253 | 0.9998 | 0.501 | 1.00 |
| 6 | Luteolin | 1.00~502 | y=0.00707x+0.012 | 0.9996 | 1.00 | 2.01 |
| 7 | Apigenin | 0.201~502 | y=0.0114x+0.0127 | 0.9988 | 0.201 | 2.01 |
| 8 | Chrysin | 0.200~501 | y=0.00624x+0.00177 | 0.9978 | 0.200 | 0.501 |

## **Accuracy and Precision**

The precision and accuracy of the method were evaluated using quality control (QC) samples at four concentration levels: lower limit of quantification (LLOQ), low (QCL), medium (QCM), and high (QCH), prepared according to the sample preparation procedure described above. Intra-day precision and accuracy were assessed by analyzing six replicates of each QC level within a single analytical run. Inter-day precision and accuracy were determined by repeating the same analysis on three consecutive days (n = 3).

As shown in Table S3, all eight analytes demonstrated excellent performance: Intra-day and inter-day RSD values were ≤15%，Accuracy (recovery) ranged from 85.0% to 115.0% across all concentration levels. These results confirm that the developed HPLC–MS/MS method is sufficiently precise, accurate, and reproducible for the quantitative analysis of flavonoids in skin tissue.

**TABLE S3** Intra-day and inter-day precision and accuracy of the eight target analytes in rat skin homogenate (n = 6 for intra-day; n = 3 for inter-day).

| Analyte | Concentration of standard（(ng/mL） | Intra-day（n=6） | | Inter-day（n=3） | |
| --- | --- | --- | --- | --- | --- |
|  |  | Degree of accuracy（%） | RSD（%） | Degree of accuracy（%） | RSD（%） |
| Esculetin | 4.02 | 102.36±7.53 | 7.36 | 97.73±4.75 | 4.86 |
|  | 10.0 | 99.98±6.98 | 6.98 | 93.66±5.49 | 5.86 |
|  | 201 | 107.30±3.35 | 3.13 | 106.99 ± 0.29 | 0.27 |
|  | 402 | 106.34±4.31 | 4.06 | 104.63±2.23 | 2.13 |
| Schaftoside | 0.500 | 99.90±4.55 | 4.56 | 97.64±2.02 | 2.07 |
|  | 1.00 | 106.27±6.05 | 5.69 | 104.69±4.10 | 3.92 |
|  | 200 | 103.67±4.78 | 4.61 | 94.48±7.98 | 8.45 |
|  | 400 | 105.04±6.74 | 6.42 | 94.76±8.92 | 9.41 |
| Luteolin-7-O-glucuronide | 0.402 | 100.33±8.90 | 8.87 | 100.83±1.63 | 1.62 |
|  | 1.01 | 96.53±8.24 | 8.53 | 96.36±0.36 | 0.38 |
|  | 201 | 102.90±5.88 | 5.72 | 97.83±4.83 | 4.94 |
|  | 402 | 107.67±6.57 | 6.10 | 101.98±5.01 | 4.92 |
| Luteolin-7-O-glucoside | 0.400 | 99.88±6.71 | 6.72 | 101.46±1.45 | 1.42 |
|  | 1.00 | 96.90±5.74 | 5.93 | 97.10±3.10 | 3.20 |
|  | 200 | 100.17±4.68 | 4.67 | 100.45±0.55 | 0.55 |
|  | 400 | 101.29±8.59 | 8.48 | 102.64±1.25 | 1.21 |
| Apigenin-7-O-glucuronide | 0.501 | 98.80±2.92 | 2.96 | 97.80±1.09 | 1.12 |
|  | 1.00 | 101.42±3.67 | 3.62 | 99.79±1.1.18 | 1.18 |
|  | 200 | 103.25±8.31 | 8.04 | 103.25±0.25 | 0.24 |
|  | 401 | 104.36±6.80 | 6.52 | 103.89±0.78 | 0.75 |
| Luteolin | 1.00 | 98.27±7.98 | 8.12 | 98.97±1.27 | 1.28 |
|  | 2.01 | 102.82±7.51 | 7.30 | 96.08±5.94 | 6.18 |
|  | 201 | 99.59±8.30 | 8.34 | 97.63±1.95 | 2.00 |
|  | 401 | 100.87±8.57 | 8.50 | 98.24±2.31 | 2.35 |
| Apigenin | 0.201 | 98.01±9.04 | 9.22 | 98.65±0.96 | 0.97 |
|  | 0.502 | 99.97±2.30 | 2.30 | 99.59±0.33 | 0.33 |
|  | 201 | 105.06±4.83 | 4.60 | 97.34±6.69 | 6.87 |
|  | 402 | 103.44±4.42 | 4.27 | 97.78±4.91 | 5.02 |
| Chrysin | 0.200 | 103.75±6.65 | 6.41 | 103.75±1.42 | 1.37 |
|  | 0.501 | 102.69±3.67 | 3.58 | 103.78±1.45 | 1.39 |
|  | 200 | 100.50±7.69 | 7.65 | 100.82±0.35 | 0.35 |
|  | 401 | 104.57±7.25 | 6.93 | 101.63±2.89 | 2.84 |

## **Matrix Effect and Recovery**

Matrix effect and extraction recovery were evaluated using quality control (QC) samples at low (QCL), medium (QCM), and high (QCH) concentrations (n = 6).

As summarized in Table S4, the extraction recoveries for the eight analytes ranged from 85.22% to 114.90%, indicating efficient and consistent recovery with minimal analyte loss during protein precipitation. The matrix effects ranged from 86.53% to 114.78%, demonstrating negligible ion suppression or enhancement from skin homogenate components. Both the analytes and the internal standard exhibited consistent behavior across all QC levels, confirming the robustness of the sample preparation and analytical method.

**TABLE S4** Matrix effects and extraction recovery of the eight target analytes in rat skin homogenate (n = 6).

| Analyte | Concentration of standard（(ng/mL） | Matrix effect（%） | RSD（%） | Recovery rate（%） | RSD（%） |
| --- | --- | --- | --- | --- | --- |
| Esculetin | 10.0 | 94.92±8.39 | 8.84 | 90.03±2.19 | 2.44 |
|  | 201 | 94.21±3.23 | 3.43 | 94.85±7.38 | 7.78 |
|  | 402 | 97.87±11.88 | 12.13 | 95.63±4.04 | 4.22 |
| Schaftoside | 1.00 | 103.11±6.42 | 6.23 | 102.58±7.06 | 6.88 |
|  | 200 | 109.30±3.80 | 3.48 | 103.37±8.75 | 8.46 |
|  | 400 | 109.34±4.08 | 3.73 | 107.46±7.44 | 6.93 |
| Luteolin-7-O-glucuronide | 1.01 | 100.14±11.37 | 11.35 | 100.83±7.77 | 7.71 |
|  | 201 | 107.78±5.22 | 4.48 | 97.05±7.76 | 8.00 |
|  | 402 | 101.51±9.91 | 9.76 | 103.83±6.07 | 5.85 |
| Luteolin-7-O-glucoside | 1.00 | 104.95±7.44 | 7.09 | 99.99±6.03 | 6.03 |
|  | 200 | 106.47±4.33 | 4.07 | 90.51±3.00 | 3.31 |
|  | 400 | 103.59±10.03 | 9.69 | 105.38±7.13 | 6.76 |
| Apigenin-7-O-glucuronide | 1.00 | 107.18±5.26 | 4.91 | 100.43±4.04 | 4.02 |
|  | 200 | 102.97±6.99 | 6.78 | 102.41±7.67 | 7.49 |
|  | 401 | 108.81±4.15 | 3.82 | 105.52±9.00 | 8.53 |
| Luteolin | 2.01 | 97.33±4.32 | 4.44 | 99.75±2.23 | 2.23 |
|  | 201 | 97.05±5.60 | 5.77 | 98.11±5.30 | 5.40 |
|  | 401 | 101.47±8.76 | 8.63 | 107.56±8.76 | 8.63 |
| Apigenin | 0.502 | 100.52±4.07 | 4.05 | 100.06±3.30 | 3.30 |
|  | 201 | 107.50±7.28 | 6.77 | 101.89±2.86 | 2.81 |
|  | 402 | 97.04±8.78 | 9.05 | 105.90±8.78 | 9.05 |
| Chrysin | 0.501 | 96.44±7.97 | 8.27 | 100.30±2.20 | 2.19 |
|  | 200 | 99.52±10.85 | 10.91 | 97.00±10.87 | 11.11 |
|  | 401 | 94.87±7.86 | 8.29 | 95.27±10.05 | 10.55 |

## **Stability**

The stability of the eight analytes in rat skin homogenate was evaluated under various storage and handling conditions using quality control (QC) samples at low (QCL), medium (QCM), and high (QCH) concentrations (n = 6 for each condition): stored at room temperature for 6 hours, at 4°C for 12 hours, subjected to three freeze-thaw cycles at -80°C, frozen at -80°C for 30 days, and placed in an automatic sampler for 24 hours after treatment. As summarized in Table S5, all eight analytes demonstrated excellent stability across all tested conditions, with RSD values <15% and recoveries within the acceptable range. These results confirm that the analytes in skin homogenate are stable under routine laboratory handling, processing, and storage conditions, supporting the reliability of the bioanalytical method for pharmacokinetic and tissue distribution studies.

**TABLE S5** Stability of the eight target analytes in rat skin homogenate under various storage and handling conditions (n = 6).

| Analyte | Concentration of standard（(ng/mL） | RSD（%） | | | | |
| --- | --- | --- | --- | --- | --- | --- |
|  |  | Room temperature for 6 hours | Place at 4℃ for 12 h | Subjected to three freeze-thaw cycles at -80°C | Frozen at -80°C for 30 days | Placed in an automatic sampler for 24 hours after treatment |
| Esculetin | 10.0 | 3.66 | 6.98 | 2.11 | 6.72 | 4.50 |
|  | 201 | 6.54 | 4.55 | 4.11 | 2.06 | 6.80 |
|  | 402 | 3.95 | 4.36 | 6.82 | 7.14 | 3.36 |
| Schaftoside | 1.00 | 5.35 | 5.69 | 8.64 | 3.10 | 3.44 |
|  | 200 | 4.83 | 7.20 | 5.02 | 5.12 | 4.35 |
|  | 400 | 2.71 | 5.06 | 5.79 | 3.67 | 3.57 |
| Luteolin-7-O-glucuronide | 1.01 | 7.68 | 8.53 | 7.82 | 6.87 | 6.55 |
|  | 201 | 1.50 | 4.79 | 6.48 | 6.21 | 8.32 |
|  | 402 | 8.39 | 6.72 | 6.33 | 4.81 | 5.38 |
| Luteolin-7-O-glucoside | 1.00 | 6.13 | 5.93 | 7.72 | 6.58 | 5.21 |
|  | 200 | 7.09 | 5.75 | 8.31 | 6.65 | 6.20 |
|  | 400 | 5.44 | 4.17 | 7.30 | 7.40 | 6.95 |
| Apigenin-7-O-glucuronide | 1.00 | 9.53 | 3.62 | 7.38 | 7.76 | 6.06 |
|  | 200 | 5.32 | 6.75 | 5.78 | 6.11 | 3.44 |
|  | 401 | 8.29 | 6.47 | 8.27 | 3.76 | 3.85 |
| Luteolin | 2.01 | 4.78 | 7.30 | 7.30 | 8.38 | 5.83 |
|  | 201 | 6.47 | 7.96 | 8.92 | 3.91 | 6.96 |
|  | 401 | 6.99 | 5.42 | 6.53 | 5.34 | 4.66 |
| Apigenin | 0.502 | 3.11 | 2.30 | 3.67 | 5.84 | 4.94 |
|  | 201 | 7.09 | 8.60 | 6.51 | 7.69 | 4.33 |
|  | 402 | 10.85 | 8.80 | 6.26 | 8.00 | 5.60 |
| Chrysin | 0.501 | 3.80 | 3.58 | 7.30 | 3.65 | 1.72 |
|  | 200 | 7.45 | 8.86 | 5.69 | 10.91 | 7.13 |
|  | 401 | 5.80 | 6.84 | 5.10 | 11.47 | 8.99 |

## **Dilute Effects**

To evaluate the reliability of sample dilution—particularly for samples with analyte concentrations exceeding the upper limit of quantification (ULOQ)—a high-concentration calibration standard (CS) containing all eight analytes at 2000 ng/mL was prepared (n = 6). This solution was spiked into blank rat skin homogenate and subsequently diluted 10-fold with additional blank homogenate to yield a final nominal concentration of 200 ng/mL for each analyte, with the internal standard (digoxin) maintained at 100 µg/mL.

As shown in Table S6, all eight analytes demonstrated excellent dilution integrity: Accuracy ranged from 85.0% to 115.0%, Intra-batch precision (RSD) was ≤15% for all compounds. These results confirm that the method supports reliable 10-fold dilution of skin homogenate samples without compromising analytical performance, ensuring accurate quantification of high-concentration samples that fall above the ULOQ.

**TABLE S6** Dilution reliability of the eight target analytes in rat skin homogenate following 10-fold dilution (n = 6).

| Analyte | Diluted concentration（(ng/mL） | Degree of accuracy（%） | RSD（%） |
| --- | --- | --- | --- |
| Esculetin | 201 | 104.39±5.79 | 5.54 |
| Schaftoside | 200 | 94.33±9.16 | 9.71 |
| Luteolin-7-O-glucuronide | 201 | 98.76±5.91 | 5.98 |
| Luteolin-7-O-glucoside | 200 | 101.00±7.01 | 6.94 |
| Apigenin-7-O-glucuronide | 200 | 104.25±4.52 | 4.34 |
| Luteolin | 201 | 104.31±6.13 | 5.88 |
| Apigenin | 201 | 96.35±5.25 | 5.45 |
| Chrysin | 200 | 101.92±7.18 | 7.04 |

## **Pharmacokinetics results**

Pharmacokinetic parameters of the seven flavonoids in mouse skin were calculated using non-compartmental analysis (DAS 2.0). Results are presented as mean ± SD (n = 8) in Table S7.

**TABLE S7** Pharmacokinetic parameters of seven flavonoid components from *Cayratia japonica* ointment (CJO) in mouse skin following topical administration (n = 8).

| Pharmacokinetic parameters | Schaftoside | Luteolin-7-O-glucoside | Luteolin-7-O-glucuronide | Apigenin-7-O-glucuronide | Luteolin | Apigenin | Chrysin |
| --- | --- | --- | --- | --- | --- | --- | --- |
| C_max_(ng/g) | 449.17±122.92 | 140.90±48.73 | 109.06±66.78 | 28.30±11.46 | 947.29±285.90 | 3620.83±1027.12 | 3.34±1.01 |
| T_max_(h) | 10.25±1.98 | 7.75±2.49 | 9.63±3.38 | 11.50±0.93 | 7.50±2.98 | 8.50±3.34 | 8.25±2.71 |
| T_1/2_(h) | 4.55±3.18 | 6.18±4.22 | 2.81±1.29 | 2.08±0.60 | 7.13±6.07 | 4.81±2.44 | 4.48±1.99 |
| AUC_0−t_(ng·h/g) | 2298.14±538.09 | 736.91±215.35 | 336.45±110.73 | 80.49±21.43 | 5195.06±1318.86 | 18327.45±4253.26 | 16.33±4.87 |
| AUC_0−∞_(ng·h/g) | 4293.80±1192.20 | 1456.62±832.40 | 725.12±312.88 | 163.36±56.62 | 11878.05±7924.37 | 37799.78±5528.09 | 33.68±6.28 |
| Vz/F(g ) | 810.70±664.98 | 3404.25±1871.59 | 3896.18±3514.28 | 11057.43±7745.08 | 418.95±163.62 | 92.08±47.32 | 99858.26±47720.83 |
| CLz/F(g h^−1^ ) | 124.55±34.48 | 476.40±281.33 | 855.61±468.88 | 3494.23±1476.16 | 61.54±41.68 | 13.45±1.80 | 15345.07±3086.32 |
| MRT_0-t_(h) | 7.55±0.27 | 7.22±0.64 | 8.38±1.14 | 8.88±0.64 | 7.28±0.49 | 7.53±0.55 | 7.68±0.54 |
| MRT_0-∞_(h) | 14.18±7.74 | 13.16±6.12 | 13.24±8.97 | 12.76±7.23 | 15.17±7.98 | 12.86±3.80 | 12.28±3.69 |

C_max_: Maximum observed concentration in skin homogenate; T_max_: Time to reach C_max_; T_1/2_: Terminal elimination half-life; AUC_0−t_: Area under the concentration–time curve from 0 h to 12 h; AUC_0-∞_: Extrapolated total AUC; V_z/F_: Apparent distribution volume; CL_z/F_: Time-Weighted Average Body Clearance Rate; MRT: Average residence time. All measurements are reported as mean ± standard deviation (SD).

1. **Validation of Molecular Docking Protocol Using Pharmacological Control Compounds**

To validate the specificity and reliability of the molecular docking protocol employed in this study, tofacitinib (a clinically approved JAK1/STAT3 inhibitor) was selected as the positive control, and chrysin (a structurally analogous flavonoid lacking the 4'-hydroxyl group of apigenin) was chosen as the negative control. All docking simulations were conducted using consistent parameters (grid box centered on the active site of JAK1/STAT3, ligand preparation with Gasteiger charges, and AutoDock Vina as the scoring function) to ensure experimental reproducibility. The binding energy values (expressed in kJ/mol) and key hydrogen bond interactions in table S8:

**TABLE S8** Molecular Docking Parameters and Binding Affinities of Apigenin and Control Molecules to JAK1 and STAT3

| Molecule | JAK1 Binding Energy (kcal /mol) | STAT3 Binding Energy (kcal /mol) | Key Hydrogen Bond Interactions |
| --- | --- | --- | --- |
| Apigenin | −8.2 | −8.4 | JAK1: ARG-1007, ASP-921, GLU-925; STAT3: ARG-609, SER-611, LYS-591 |
| Tofacitinib (Positive Control) | −8.9 | −9.2 | JAK1: ASP-921, GLU-925; STAT3: ARG-609, SER-611, LYS-591 |
| Chrysin (Negative Control) | −6.8 | −7.0 | JAK1: ASP-921 (weak); STAT3: Weak or no hydrogen bonds with key residues |

Notes: Binding energy values represent the mean of three independent docking replicates. More negative values indicate stronger binding affinity between the ligand and the target protein.

Hydrogen bond interactions were identified using PyMOL v2.5, with a distance cutoff of 3.5 Å and an angle cutoff of 120°.

Chrysin was selected as the negative control due to its structural similarity to apigenin and the reported loss of anti-inflammatory activity caused by the absence of the 4'-hydroxyl group (Caporali et al., 2022).

The binding pattern of tofacitinib is consistent with its well-documented mechanism of action targeting the active site of JAK1/STAT3, which confirms the validity of the docking protocol used in this study.
